# *Snrpb* is required in murine neural crest cells for proper splicing and craniofacial morphogenesis

**DOI:** 10.1242/dmm.049544

**Published:** 2022-06-23

**Authors:** Sabrina Shameen Alam, Shruti Kumar, Marie-Claude Beauchamp, Eric Bareke, Alexia Boucher, Nadine Nzirorera, Yanchen Dong, Reinnier Padilla, Si Jing Zhang, Jacek Majewski, Loydie A. Jerome-Majewska

**Affiliations:** 1 Research Institute of the McGill University Health Centre at Glen Site, Montreal, QC H4A 3J1, Canada; 2 Department of Human Genetics, McGill University, Montreal, QC H3A 0G1, Canada; 3 Department of Anatomy and Cell Biology, McGill University, Montreal, QC H3A 2B2, Canada; 4 Department of Pediatrics, McGill University, Montreal, QC H4A 3J1, Canada

**Keywords:** Cerebrocostomandibular syndrome, *SNRPB*, Splicing, Neural crest cells, Craniofacial

## Abstract

Heterozygous mutations in *SNRPB*, an essential core component of the five small ribonucleoprotein particles of the spliceosome, are responsible for cerebrocostomandibular syndrome (CCMS). We show that *Snrpb* heterozygous mouse embryos arrest shortly after implantation. Additionally, heterozygous deletion of *Snrpb* in the developing brain and neural crest cells models craniofacial malformations found in CCMS, and results in death shortly after birth. RNAseq analysis of mutant heads prior to morphological defects revealed increased exon skipping and intron retention in association with increased 5′ splice site strength. We found increased exon skipping in negative regulators of the P53 pathway, along with increased levels of nuclear P53 and P53 target genes. However, removing *Trp53* in *Snrpb* heterozygous mutant neural crest cells did not completely rescue craniofacial development. We also found a small but significant increase in exon skipping of several transcripts required for head and midface development, including *Smad2* and *Rere*. Furthermore, mutant embryos exhibited ectopic or missing expression of *Fgf8* and *Shh*, which are required to coordinate face and brain development. Thus, we propose that mis-splicing of transcripts that regulate P53 activity and craniofacial-specific genes contributes to craniofacial malformations.

This article has an associated First Person interview with the first author of the paper.

## Abstract (precis)

**Summary:** We report the first mouse model for cerebrocostomandibular syndrome, showing that mis-splicing of transcripts that regulate P53 activity and craniofacial-specific genes contributes to craniofacial malformations.

## INTRODUCTION

Ninety-five percent of human pre-mRNAs are alternatively spliced to generate multiple mRNAs (Chen and Manley, 2009; Nilsen and Graveley, 2010), thus increasing the number and diversity of proteins expressed from the human genome. The major spliceosome or U2-dependent spliceosome catalyzes 99% of RNA splicing reactions in human (Wickramasinghe et al., 2015), while the minor or U12-dependent spliceosome is responsible for splicing of ∼700 minor introns in 666 genes (Olthof et al., 2019). The major spliceosome is composed of U1, U2, U5 and U4/U6 small nuclear ribonucleoproteins (snRNPs), named for their core-associated small nuclear RNAs (snRNAs). *SNRPB* encodes SmB and SmB′, which are core components of the spliceosome. SmB/B′ help to form the heptameric ring on the U snRNAs of the five snRNPs of the major spliceosome. Several groups have reported heterozygous mutations in *SNRPB* in patients with cerebrocostomandibular syndrome (CCMS; OMIM #117650) (Lynch et al., 2014; Bacrot et al., 2015; Tooley et al., 2016). CCMS patients have rib gaps and narrow chests, and craniofacial defects such as malar hypoplasia and micrognathia, with variable expressivity (Beauchamp et al., 2020) and incomplete penetrance.

In addition to the two coding transcripts, SmB and SmB′, *SNRPB* also encodes a third premature termination codon (PTC)-containing transcript that is predicted to undergo nonsense-mediated decay (Saltzman et al., 2011). Most mutations found in CCMS patients increase inclusion of the PTC-containing alternative exon 2, leading to no change in or reduced levels of the coding transcripts in a patient's fibroblasts (Bacrot et al., 2015; Lynch et al., 2014). However, although it is presumed that increased expression of the PTC-containing transcript leads to reduced levels of SmB/SmB′ in all CCMS patients, reduced levels of SNRPB protein have not been reported in any CCMS patient cells. We postulated that a mouse model carrying mutation in *Snrpb* can be used to understand the role of *Snrpb* during embryogenesis and gain insight into the pathophysiology of CCMS.

Towards this goal, we generated a conditional mutant mouse line with *loxP* sequences flanking the genomic region that encompasses exons 2 and 3 of *Snrpb*. Using *β-actin-Cre*, we showed that widespread heterozygous deletion (*Snrpb^+/−^*) of these exons reduced levels of *Snrpb* and resulted in embryonic arrest by embryonic day (E)9.5. To investigate the role of *Snrpb* specifically during craniofacial development, we used *Wnt1-Cre2* to generate *Snrpb* heterozygosity in the developing brain and neural crest cells (*Snrpb^ncc+/−^*). A subset of these embryos survived to birth and died shortly after. Most *Snrpb^ncc+/−^* mutant embryos died between E17.5 and birth, with brain and craniofacial defects of variable expressivity. RNA-sequencing (RNAseq) analysis of the E9.0 embryonic heads of *Snrpb^ncc+/−^* mutants, before morphological defects were apparent, revealed a significant increase in differential splicing events (DSEs), but few differentially expressed genes (DEGs). Pathway analysis indicated that these DEGs were associated with the spliceosome and the P53 (also known as TP53) pathway. However, although nuclear P53 and apoptosis were increased in *Snrpb^ncc+/−^* embryos, reducing levels of P53 genetically in the neural crest did not prevent craniofacial defects in these mutants. Intriguingly, a number of DSEs were found in genes important for craniofacial development. Furthermore, the expression of *Shh* and *Fgf8*, which forms the facial organizer, was disrupted in the craniofacial region. Our findings support disrupted splicing as the major driver of abnormalities in *Snrpb* mutant embryos. We suggest that abnormal splicing of genes important for craniofacial development results in an additive effect that disrupts morphogenesis of the head and brain.

## RESULTS

### Mouse embryos with deletion of exons 2 and 3 of *Snrpb* (*Snrpb^+/−^*) have reduced levels of *Snrpb* and die post-implantation

**Fig. 1.**
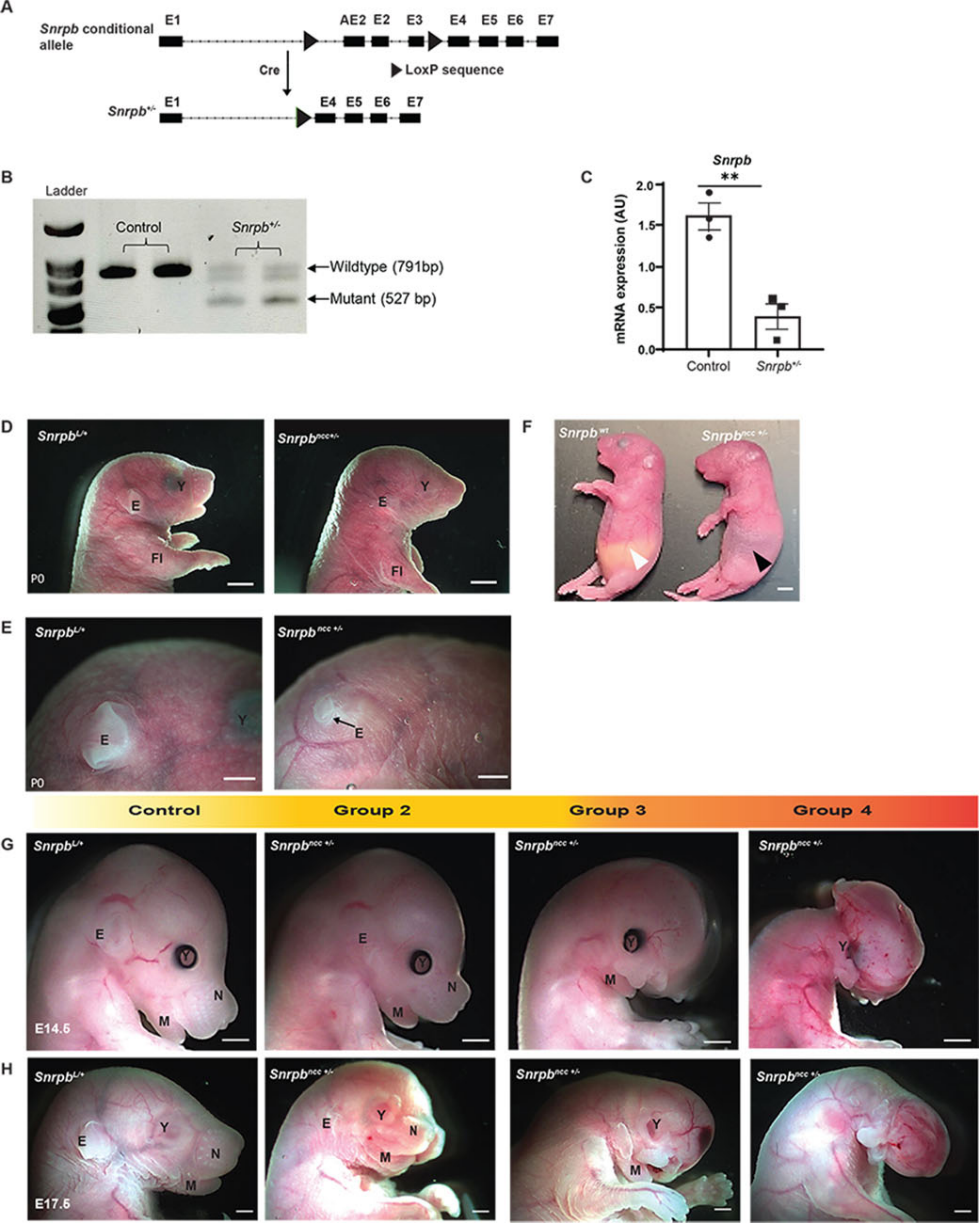
**Deletion of exons 2-3 of *Snrpb* results in reduced *Snrpb* level and craniofacial malformations of varying severity.** (A) Schema of the conditional *Snrpb^loxP/+^* allele generated using CRISPR/Cas9 and the deletion generated in the presence of *Cre*. (B) Deletion of the *loxP*-flanked exons 2-3 in *Snrpb* produces a shorter transcript of 527 bp. (C) Quantitative RT-PCR showing a significant decrease in *Snrpb* level in E8.5 heterozygous mutant embryos (**P<0.01, unpaired, two-tailed *t*-test). (D) P0 *Snrpb^ncc+/−^* (*Snrpb^loxP/+^*; *Wnt-1Cre2^tg/+^*) pups have an abnormally shaped head, micrognathia and abnormal outer ears. (E) Higher-magnification images showing a hypoplastic pinna (E) in the *Snrpb^ncc+/−^* mutant (black arrow) compared to control (*Snrpb^L^*^/+^). (F) *Snrpb^ncc+/−^* mutant pups lack a milk spot in their stomach (black arrowhead), which is visible in the *Snrpb* wild-type (WT) littermate (white arrowhead). (G,H) Representative images of *Snrpb* WT (*Snrpb^L^*^/+^) and *Snrpb^ncc+/−^* mutant embryos at E14.5 (G) and E17.5 (H). *Snrpb^ncc+/−^* mutant embryos show a range of craniofacial malformations: Group 2 had abnormal outer ear, and cranial and mandibular hypoplasia; Group 3 had nasal clefts; Group 4 showed severe abnormalities including absence of the head and face. AU, arbitrary units; E, ear; Fl, forelimb; M, mandible; N, nose; Y, eye. Scale bars: 1 mm.

To test whether reduced levels of *Snrpb* in mouse recapitulates abnormalities found in CCMS patients, we first generated a conditional mutant mouse line with *loxP* sequences in intron 1 and intron 3 of the gene. We then used *β-actin*-*Cre* to delete the *loxP*-flanked region – exon 2, the PTC encoding alternative exon 2 and exon 3 of *Snrpb* (Fig. 1A) – to produce *Snrpb* heterozygous mice (*Snrpb^+/−^*). However, no *Snrpb^+/−^* mice were found at birth [postnatal day (P)0] or at weaning, indicating that these mutants died before birth (*n*=22, three litters, *P*=0.04). We confirmed that *Cre*-mediated deletion of the *loxP*-flanked region generated a shorter *Snrpb* transcript of 527 bp (Fig. 1B) and resulted in a statistically significant 70% reduction in levels of *Snrpb* in E8.5 *Snrpb^+/−^* embryos (*P*=0.0052, unpaired, two-tailed *t*-test) (Fig. 1C). Thus, deletion of exons 2-3 of *Snrpb* led to a significant reduction in *Snrpb* levels in heterozygous mutant embryos. Our data indicate that the amount of functional protein expressed by a single wild-type (WT) allele of *Snrpb* was insufficient for embryonic growth and survival post-implantation.

### *Snrpb* is required in the neural crest cells for craniofacial development and postnatal survival

We next used a *Wnt1-Cre2* transgene to delete *Snrpb* in the neural tube and neural crest cells (*Snrpb^ncc+/−^*) to examine its role during craniofacial development. No *Snrpb^ncc+/−^* mutant pups were found at P1 and P21 (*P*>0.0001, chi-square). At P0, we recovered five heterozygous *Snrpb^ncc+/−^* pups from six litters (*n*=47). Most had no visible milk spots, indicating that they failed to feed (*n*=4/5). One *Snrpb^ncc+/−^* pup was morphologically normal, while the rest had abnormally shaped heads, short snouts and small outer ears (*n*=4) (Fig. 1D-F). To determine when *Snrpb* is first required in neural crest cells for embryonic survival and craniofacial development, we collected and analyzed embryos from E9.0 to E17.5. *Snrpb^ncc+/−^* embryos were found at the expected Mendelian ratio until E17.5, when significantly fewer mutant embryos were found (Table 1) (*P*<0.025, chi-square test). At E14.5 and E17.5, 43% and 25% of *Snrpb^ncc+/−^* embryos were dead and undergoing resorption, respectively. Thus, a significant number of *Snrpb^ncc+/−^* embryos die between E14.5 and birth.

**Table 1.**
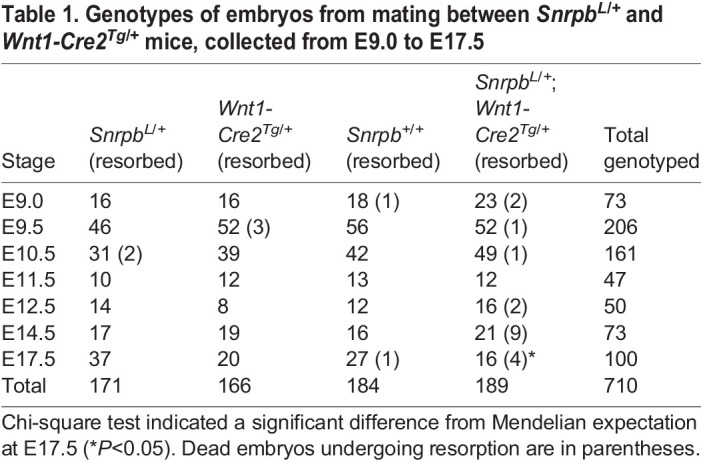
Genotypes of embryos from mating between *Snrpb^L/+^* and *Wnt1-Cre2^Tg/+^* mice, collected from E9.0 to E17.5

We found that, at E9.0, *Snrpb^ncc+/−^* embryos with 13 or fewer somites were indistinguishable from control (*Snrpb^+/+^* or *Wnt^tg/+^*) littermates (Table 2; Fig. S1A). However, morphological abnormalities were first apparent in live *Snrpb^ncc+/−^* embryos at E9.5. At this stage, 35% of *Snrp^ncc+/−^* mutants (*n*=18), exhibited hypoplasia of the midbrain and hindbrain. At E10.5, 74% of mutant E10.5 embryos (*n*=43) also showed hypoplasia of the frontonasal, maxillary and mandibular prominences, and the pharyngeal arches, and a smaller midbrain and hindbrain (Table 2; Fig. S1B,C). E11.5 *Snrpb^ncc+/−^* embryos (*n*=12) could be sorted into three groups based on their shared phenotypes. We assigned the 17% of embryos that were morphologically normal and indistinguishable from controls to group 1/normal (*n*=2); the 17% of mutants with hypoplasia of the developing brain, face and head to group 2 (*n*=2); and the remaining 66% to group 3 (*n*=8). Abnormalities found in group 3 included hypoplasia of the midbrain, swelling in the forebrain, subepidermal swelling, absence of the frontonasal and the maxillary prominences, and a hypoplastic mandibular arch (Table 2; Fig. S1D). At E12.5, 25% were morphologically normal (*n*=4; group 1). Morphologically abnormal mutants at this stage were classified as group 2 (19%; *n*=3) or group 3 (25%; *n*=4). Mutants in group 2 exhibited clefts in the frontonasal prominence and the mandible, while those in group 3 had hypoplasia of the midbrain, an abnormal forebrain, and cleft of the hypoplastic frontonasal and maxillary prominences (Table 2; Fig. S1E). A fourth phenotypic group constituting 19% of *Snrpb^ncc+/−^* embryos was found (*n*=3) at E12.5. Embryos in this group showed absence of the ventral portion of the head and face, edema in the head and a hypoplastic mandibular arch (Table 2; Fig. S1E, rightmost image). At E14.5, morphologically normal, group 1 *Snrpb^ncc+/−^* embryos comprised 8% of live mutant embryos (*n*=1). Mutant embryos in group 2 (*n*=3) had a hypoplastic pinna, a dome-shaped head and nasal clefts; and those in group 3 (*n*=4), showed hypoplasia and cleft of the frontonasal, maxilla and mandibular regions, and subepidermal edema (Table 2, Fig. 1G; Fig. S1F). *Snrpb^ncc+/−^* embryos in group 4 (*n*=4) showed the most severe abnormalities (Table 2, Fig. 1G; Fig. S1F), and were missing the ventral portion of the head and face. At E17.5, phenotypically normal group 1 embryos were not found. Half of the mutant embryos found alive were classified as group 2 (*n*=6), and the remainder were in groups 3 (*n*=4) and 4 (*n*=2) (Table 2, Fig. 1H; Fig. S1G). Thus, WT levels of *Snrpb* are critical in the neural crest cells from E9.5 onwards for normal development of the head and face.

**Table 2.**
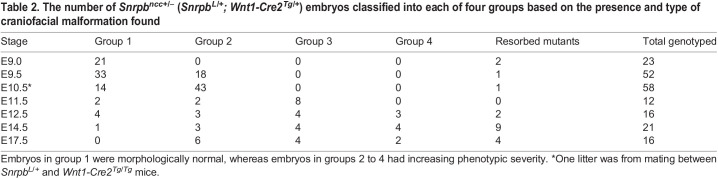
The number of *Snrpb^ncc+/−^* **(*****Snrpb***^***L/+***^***; Wnt1-Cre2***^***Tg/+***^**)** embryos classified into each of four groups based on the presence and type of craniofacial malformation found

### *Snrpb* is required in neural crest cells for proper differentiation of both mesoderm and neural crest cell-derived cartilage and bones

**Fig. 2.**
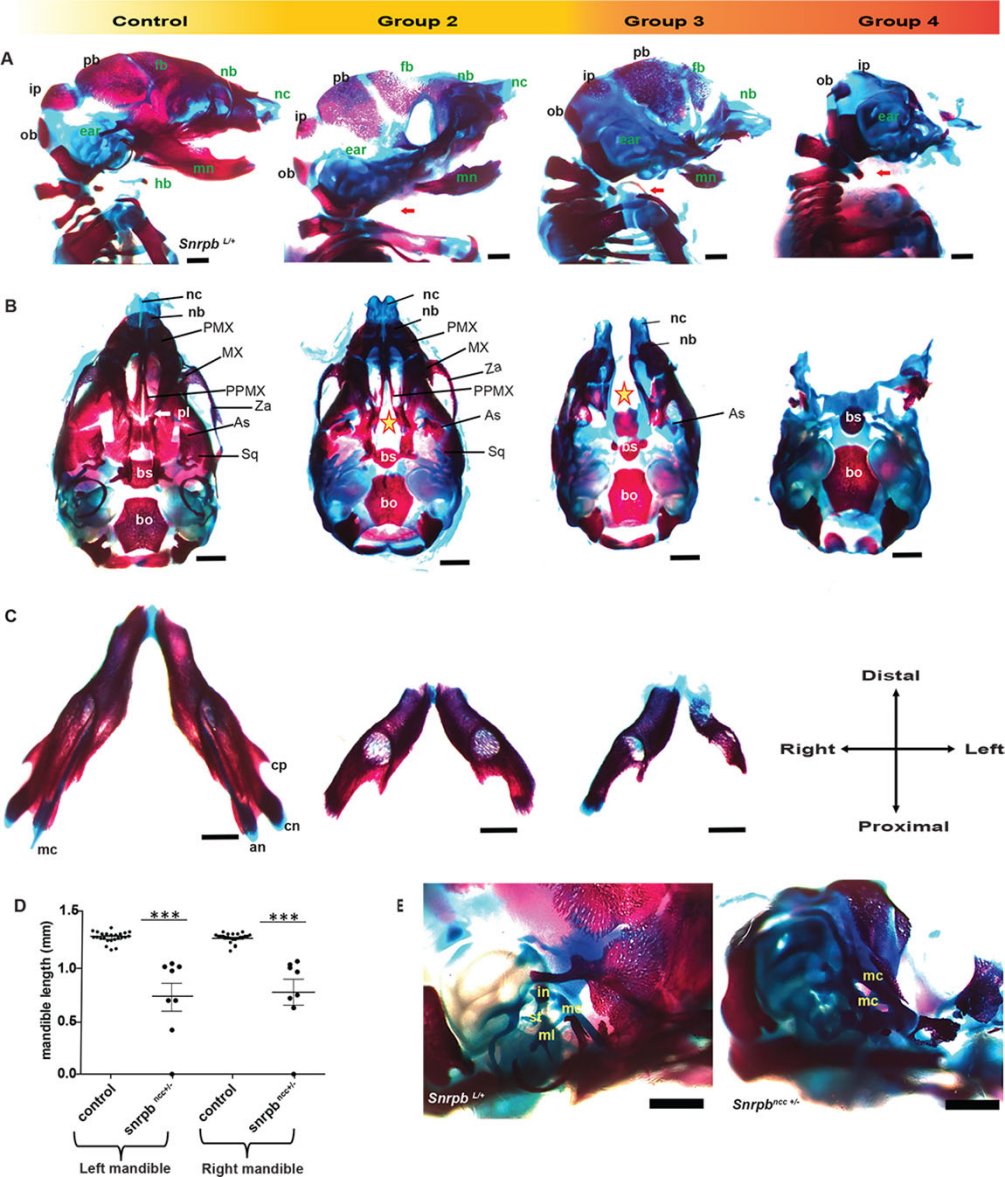
**Craniofacial cartilage and bones of *Snrpb^ncc+/−^* embryos are hypoplastic or missing.** (A,B) Representative images of Alcian Blue- and Alizarin Red-stained E17.5 normal (*Snrpb^wt^* or *Snrpb^L/+^*) and *Snrpb^ncc+/−^* mutant embryos showing craniofacial abnormalities of varying penetrance. (A) Sagittal view showing hypoplasia or absence of neural crest cell-derived bones (labeled in green font) in *Snrpb^ncc+/−^* mutants. The missing hyoid bone and tracheal cartilage are indicated by red arrows. (B) Ventral view of the skull showing palatal and maxillary clefts (stars) in group 2 and 3 *Snrpb^ncc+/−^* mutants, respectively, and the absence of the ventral craniofacial components in group 4 mutants. (C) Representative images of the lower jaw of a normal embryo and two *Snrpb^ncc+/−^* mutants, showing asymmetric mandibles with no discernable angular, coronoid or condylar processes. (D) Both left and right mandibles are significantly shorter in *Snrpb^ncc+/−^* embryos (***P<0.0001, unpaired, two-tailed *t*-test), compared to controls (*Snrpb^wt^* and *Snrpb^L/+^* embryos). (E) Representative higher-magnification images of the inner ear of a control and *Snrpb^ncc+/−^* embryo. Middle ear structures such as the stapes were absent in group 3 and 4 mutants, whereas a presumably duplicated Meckel's cartilage and ectopic structures were found in a subset. an, angular process; As, alisphenoid bone; bo, basioccipital bone; bs, basisphenoid bone; cn, condylar process; cp, coronoid process; fb, frontal bone; hb, hyoid bone; in, incus; ip, intraparietal bone; mc, Meckel's cartilage; ml, malleus; mn, mandible; MX, maxilla; nb, nasal bone; nc, nasal cartilage; ob, occipital bone; pb, parietal bone; PMX, premaxilla; pl, palatine; PPMX, palatal process of maxilla; Sq, squamous bone; st, stapes; Za, zygomatic arch. Scale bars: 500 μm.

Skeletal preparations with Alcian Blue to stain cartilage and Alizarin Red to stain bone were used to examine the development of neural crest cell derivatives in the developing head and face at E14.5, E17.5 and P0. Cartilage development in the head of the single E14.5 *Snrpb^ncc^*^+/−^ group 1 embryo found was indistinguishable from that of controls. However, in E14.5 and E17.5 *Snrpb^ncc^*^+/−^ embryos belonging to groups 2 and 3 (*n*=8), the mesoderm-derived parietal bone and the intraparietal bone – which is derived from both mesoderm and neural crest cells – were hypoplastic (Fig. 2A; Fig. S2A,B). Neural-crest derived bones such as the temporal and alisphenoid bones were missing, while the frontal and nasal bones were hypoplastic (*n*=5) (Fig. 2B; Fig. S2). Mutant embryos in these two groups also showed clefts of the nasal and pre-maxillary cartilage and bones, and the palate, as well as hypoplasia of Meckel's cartilage and its derivative, the mandible (Fig. 2C,D; Fig. S2). The zygomatic arch also failed to form in group 3 mutants (Fig. 2B). Those heterozygous mutants belonging to group 4 had hypoplasia of the basisphenoid bone and were missing neural crest cell-derived cartilage and bones that are normally found on the ventral surface of the head and face (Fig. 2B; Fig. S2). Furthermore, although the mandible formed in E14.5 and E17.5 *Snrpb^ncc+/−^* embryos in groups 2 and 3, it was both asymmetrical and bilaterally smaller compared to that of controls (Fig. 2D, unpaired, two-tailed *t*-test, *P*<0.0001). Distal ends of the jaws were abnormally shaped, while the proximal elements of the mandible such as the coronoid, condylar and angular processes were not found in mutants (Fig. 2C). Additional defects found in mutant embryos included missing tympanic ring, hypoplasia or absence of the hyoid, and missing tracheal cartilage. In addition, ectopic cartilage and bones, which could not be conclusively identified (*n*=4 of 7) (Fig. 2E; Fig. S2C), were also found in the middle ear.

At P0, the morphologically normal *Snrpb^ncc+/−^* pup/group 1, had a curved but closed premaxilla (*n*=1; Fig. S3A). Furthermore, although one of the morphologically abnormal group 2 pups had a cleft in the premaxilla and was missing the palatine shelves (*n*=1), no bony palate defects were found in the remaining mutant pups (*n*=3) (Fig. S3A). Skull defects were found in both group 1 and group 2 *Snrpb^ncc+/−^* pups. These defects included reduced size of the squamous part of the temporal bone (*n*=3), heterotopic ossification in the frontal suture (*n*=1), and a hypoplastic and asymmetric basisphenoid (*n*=3) (Fig. S3). Defects were also found in the mandible and middle ear. Meckel's cartilage and the lower jaw that forms around it were asymmetric in most of these mutants (*n*=5). Specifically, the angular process was asymmetric in the group 1 mutant with a wider angular process on one side (*n*=1) and three of the group 2 mutants (Fig. S3B). The articular surface cartilage was also absent or hypoplastic in group 1 and group 2 (*n*=2) mutants (Fig. S3B). Additionally, the condyloid and the angular processes were shortened bilaterally in one group 2 mutant (*n*=1). Middle ear defects such as absent or abnormally shaped tympanic ring and presence of ectopic ossification were found in group 1 and group 2 mutants (*n*=5; Fig. S3C). Our data indicate that *Snrpb* mutant neural crests can form cartilage but show deficiencies in ossification.

### Derivatives of cranial, cardiac and trunk neural crest cells are abnormal in *Snrpb* mutants

**Fig. 3.**
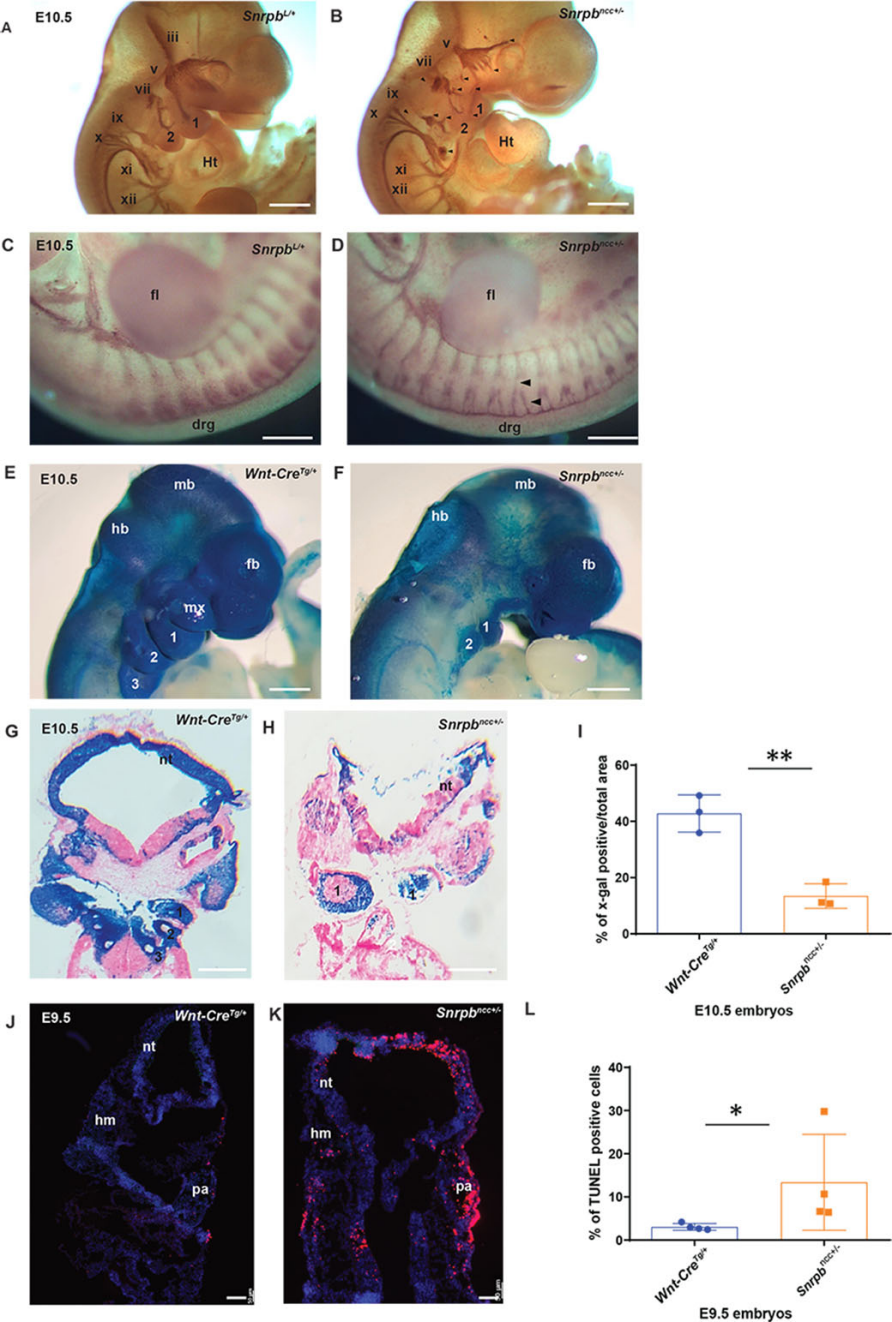
**Abnormal cranial ganglia, reduced cranial neural crest cells and a significant increase in cell death in *Snrpb^ncc+/−^* mutants.** (A,B) Representative images of E10.5 control (*Snrpb^L/+^*) (A) and *Snrpb^ncc+/−^* (B) group 2 embryos stained with antibody against neurofilament (2H3). (B) *Snrpb^ncc+/−^* mutants (*n*=2) showed abnormal projections of nerves to the pharyngeal arches (cranial ganglion v and vii) and heart (cranial ganglion ix), and absence and abnormal bundling of cranial nerves (all are indicated by black arrowheads). (C,D) Compared to controls (*Snrpb^L/+^*) (C), the dorsal root ganglia are bifurcated and reduced in mutants (black arrowheads) (D). (E-H) Representative images of X-gal-stained wholemount (E,F) and sectioned (G,H) E10.5 control (*Wnt-Cre2^tg^*^/+^) (E,G) and *Snrpb^ncc+/−^* group 2 mutant (F,H) embryos. *Snrpb^ncc+/−^* mutants show reduced X-gal staining in the craniofacial region and in the pharyngeal arches (*n*=4) compared to the control embryos (*n*=4). (I) Quantification of the area stained with X-gal showed a significant reduction in mutants (*n*=3) compared to control littermates (*n*=3) (unpaired, two-tailed *t*-test, **P<0.005). Error bars indicate s.e.m. (J,K) Representative images of sections of TUNEL-stained E9.5 control (*Snrpb^wt^* or *Snrpb^L/+^*) (J) and *Snrpb^ncc+/−^* mutant (K) embryos. (L) Quantification showed an increase in the percentage of TUNEL-positive nuclei (red in J,K) in the craniofacial region of mutants (*n*=3) (unpaired, two-tailed *t*-test, *P<0.05). Error bars indicate s.e.m. 1, 2, 3, pharyngeal arches 1, 2 and 3; drg, dorsal root ganglia; fb, forebrain; fl, forelimb; hb, hindbrain; hm, head mesenchyme; Ht, heart; mb, midbrain; mx, maxillary prominence; nt, neural tube; pa, pharyngeal arch. Scale bars: 500 μm (A-H), 50 μm (J,K).

In addition to cartilage and skeletal abnormalities, the dorsal root ganglia and cranial nerve ganglion – which form from ectodermal placodes and neural crest cells – were also abnormal in *Snrpb^ncc+/−^* mutants. Neurofilament staining of WT (Fig. 3A) and group 2 E10.5 *Snrpb^ncc+/−^* mutant embryos (Fig. 3B) showed that the cranial ganglia of mutants were reduced in size and had abnormal neuronal projection into the pharyngeal arches (*n*=2). In mutants, the ophthalmic branch of the trigeminal nerve (CN V) was reduced and did not extend over the lens, the maxillary projection appeared disorganized and missing, and the mandibular projection was reduced and appeared to have formed ventral to the first arch (Fig. 3B). In addition, an ectopic projection was found in the CN V in mutants. Furthermore, the proximal portions of the geniculate (CN VII) and vestibulo-acoustic (CN VIII) ganglia were thicker than in *Snrpb^ncc+/+^* (Fig. 3A). Similarly, the glossopharyngeal nerve (CN IX) was abnormally thicker in the proximal region before the pharyngeal arch, and had ectopic projection into pharyngeal arch 2 and reduced projection into pharyngeal arch 3 (Fig. 3B). Finally, the proximal portion of the vagus nerve (CN X) was relatively normal but had an abnormal bundle at the distal end with reduced projections into the heart. Furthermore, the dorsal root ganglia, which are derived from trunk neural crest cells, were reduced in size and bifurcated at the proximal end (Fig. 3C,D).

Micro-computed tomography (CT) scans of an E14.5 *Snrpb^ncc+/−^* embryo from group 4 revealed that the nasal septum and nasopharyngeal cavity were not formed; however, the oropharynx, tongue and pituitary gland were present (Fig. S4A). CT scans of an E17.5 WT embryo (control) and mutant embryos revealed that the aorticopulmonary septum, which is derived from cardiac neural crest cells, did not differentiate in the E17.5 group 2 *Snrpb^ncc+/−^* embryo (Fig. S4B-E). Furthermore, the thymus gland, a derivative of the third pharyngeal pouch, also failed to form in this mutant embryo (Fig. S4F). Finally, at all stages – E14.5 (*n*=1) and E17.5 (*n*=1) – the cerebral cortex was abnormally thin, and the lateral ventricles were enlarged in mutants (Fig. S4). Altogether, our morphological analysis indicates that *Snrpb* is required for the formation of structures that are derived from or induced by neural crests along the anterior–posterior axis. Our data also suggest that cardiac anomaly might contribute to death of *Snrpb^ncc+/−^* embryos and pups.

### Neural crest cells require WT levels of *Snrpb* for their survival in the craniofacial region

To track *Snrpb^ncc+/−^* heterozygous neural crest cells and their derivatives, we introduced the ROSA lacZ (Soriano, 1999) and ROSA mT/mG (Muzumdar et al., 2007) reporters into our mutant line. At E9.5, WT and *Snrpb^ncc+/−^* embryos [morphologically normal (*n*=1) and abnormal (*n*=2)] showed a comparable proportion of X-galactosidase (X-gal)-positive cells in the head and pharyngeal arches (Fig. S5A-C), with no statistically significant differences. Similarly, when the mT/mG reporter was used to visualize GFP-positive Cre-expressing cells in control (*n*=3) and morphologically normal *Snrpb^ncc+/−^* mutants (*n*=3), no difference was found (Fig. S5D-H). However, at E10.5, morphologically abnormal group 2 mutant embryos (*n*=4) showed a reduced proportion of X-gal-positive cells in the head region (Fig. 3G-I) compared to WT, and this difference was statistically significant (unpaired, two-tailed *t*-test, *P*=0.003). To determine whether reduced proliferation and/or increased apoptosis contribute to loss of X-gal-positive *Snrpb* heterozygous cells, E9.5 and E10.5 embryos were analyzed after phosphohistone H3 (PH3) immunostaining and terminal deoxynucleotidyl transferase dUTP nick end labeling (TUNEL). No significant difference in proliferation was found between E9.5 control and *Snrpb^ncc+/−^* embryos (*n*=4; *n*=3 group 1/normal and *n*=1 group 2) and E10.5 control and *Snrpb^ncc+/−^* embryos (*n*=5; *n*=1 group1/normal and *n*=4 group 2) (Fig. S5J,K). However, a statistically significant increase in TUNEL-positive cells was found in the developing head region of E9.5 *Snrpb^ncc+/−^* embryos (unpaired, two-tailed *t*-test, *P*=0.029) (*n*=4; *n*=3 group 1/normal and *n*=1 group 2) compared to controls (Fig. 3J-L). Our data indicate that *Snrpb* heterozygous cells migrate into the developing head region and the pharyngeal arches. However, a subset of these cells undergo apoptosis and are lost in mutant embryos by E10.5.

### Mutations in *Snrpb* cause an overall increase in skipped exon and intron retention

**Fig. 4.**
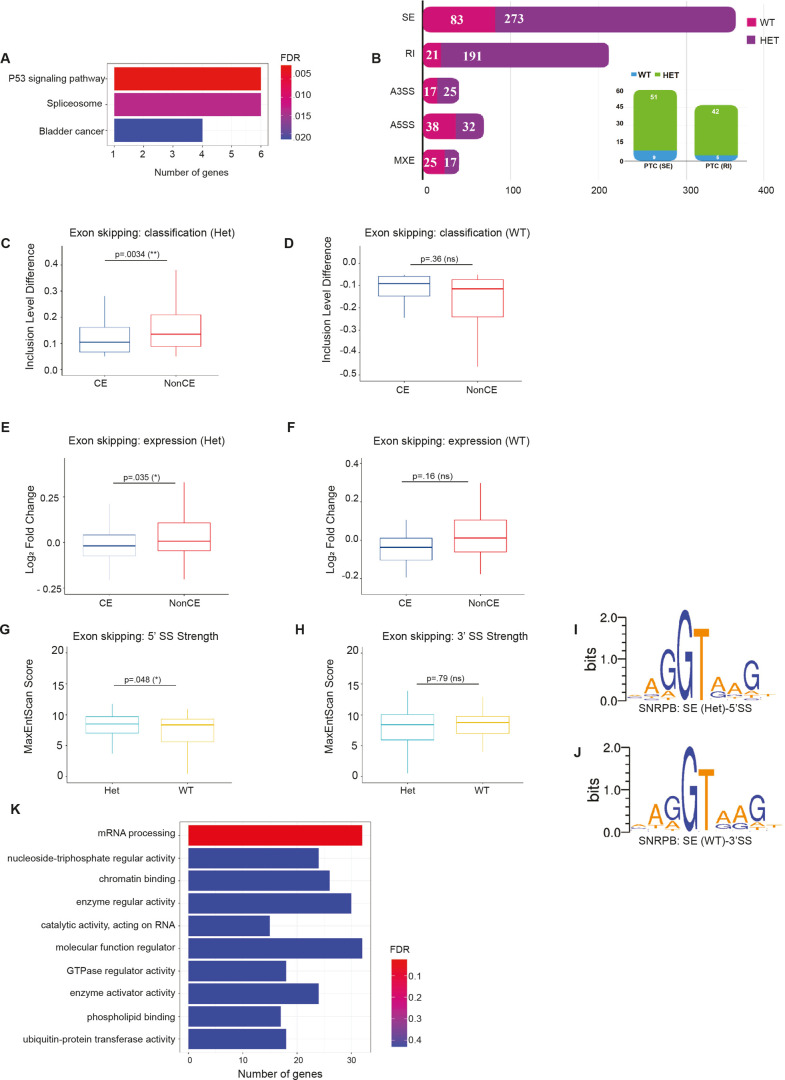
***Snrpb^ncc+/−^* mutant heads show aberrant splicing including increased exon skipping and intron retention.** (A) Differentially expressed genes (DEGs) identified in *Snrpb^ncc+/−^* (Het) mutants could be grouped into molecular pathways belonging to the P53 signaling pathway and the spliceosome. (B) A much larger number of transcripts was found to be abnormally spliced in *Snrpb^ncc+/−^* mutants compared to controls (*Snrpb^wt^*). The most abundant differentially spliced events (DSEs) were skipped exons (SEs) and retained introns (RIs). A strong tendency towards increased exon skipping and intron inclusion in the mutant samples was observed; there were more SEs (273 in Het versus 83 in WT) and RIs (191 in Het versus 21 in WT). DSEs were predicted to introduce premature termination codons (PTCs) more often in Hets compared to WT. (C-F) In Hets, exon skipping was significantly higher for non-constitutive exons (NonCE) when constitutive (CE) versus (NonCE) was examined (unpaired, two-tailed *t*-test). (G-J) An analysis of splice site (SS) strengths in DSEs revealed significantly stronger 5′ SS in mutants than in controls (*P*=0.05, unpaired, two-tailed *t*-test). (K) Pathway analysis of genes with DSEs showed that they were strongly associated with mRNA processing. ns, not significant; *P<0.05, **P<0.01.

To identify the molecular events that precede cell death, heads of E9.0 *Snrpb^ncc+/−^* embryos with 11-13 somite pairs, prior to morphological defects, were isolated and used for RNAseq analysis. Surprisingly, gene expression data did not reveal a major distinction between mutant and WT embryos, and the samples did not cluster by genotype. This was further corroborated by differential gene expression (DEG) analysis, which identified very few (76) DEGs: 50 upregulated and 26 downregulated in the mutant embryos. This low number of DEGs is consistent with the lack of a clear phenotypic distinction at this developmental stage. However, the DEGs that were identified could already be characterized into relevant molecular pathways, specifically belonging to the P53 signaling pathway and representing components of the spliceosome (Fig. 4A).

In contrast to the low number of DEGs, a large number of transcripts were found to be abnormally spliced. We identified 722 significant [false discovery rate (FDR)=0.1] differentially spliced events (DSEs) between the *Snrpb^ncc+/−^* (Het) and WT samples. The most abundant of these DSEs were skipped exons (SEs) and retained introns (RIs) (Fig. 4B). Although the high proportion of SEs could be expected based on prior alternative splicing studies, it was notable that more than 30% of the total alternative splicing events detected were RI events. We observed a strong tendency towards increased exon skipping and intron inclusion in the mutant samples; there were more SEs (273 in Het versus 83 in WT) and RIs (191 in Het versus 21 in WT) (Fig. 4B). Consistent with the absence of significant gene expression changes, DSEs in *Snrpb^ncc+/−^* embryos did not lead to significant changes in inclusion of PTC-containing exons or introns (Fig. S6A-D). However, although SEs were more likely to be alternative exons (non-constitutive) in heterozygous (*P*=0.0034) versus WT (Fig. 4C,D), expression of transcripts with SEs of constitutive exons was significantly reduced in mutants (*P*=0.0035) compared to WT (Fig. 4E,F). Those global trends in splicing are consistent with those previously found in cell culture, suggesting that SNRPB deficiency results in increased skipping of alternatively spliced exons resulting from reduced recognition of splicing signals (Correa et al., 2016).

### Increased SEs and RIs in *Snrpb* mutants are not linked to identifiable sequence features

We next investigated whether the aberrant splicing events in the mutant embryos could in fact be characterized by specific sequence features. We compared alternative events preferentially found in the mutants to two control groups: (1) events preferentially found in the WT embryos, and (2) a set of 1000 randomly chosen alternative events. Specifically, we aimed to test whether aberrant events in the mutants were associated with weaker splice signals. Although there was a very slight trend towards weaker splice site (SS) scores (MaxEntScan, Yeo and Burge, 2004) of RIs in mutant compared to *Snrpb* WT embryos, the differences were small and not statistically significant (Fig. S6E,F). In contrast, 5′ SS strength was significantly higher in *Snrpb^ncc+/−^*heterozygous compared to *Snrpb* WT embryos (Fig. 4G), whereas the 3′ SS strength was comparable between mutants and *Snrpb* WTs (Fig. 4H). We also analyzed the strength and position of predicted branch point (BP) signals (LaBranchoR, Paggi and Bejerano, 2018), but again we did not find notable differences (Fig. S6K-N), with the exception of a slight preference for a more distal BP location of mutant-specific SE events (27 bp in mutant versus 25 bp in the random set, *P*=0.026). We also looked at general base composition in SEs and RIs and no statistically significant difference was observed (Fig. S6O-R), although the GC content in retained introns was slightly increased (Fig. S6S,T). Finally, we scanned for the frequency of RNA-binding protein motifs around the mutant-specific events (rMAPS2, Hwang et al., 2020), but did not identify significant enrichment of recognition signals of known splicing factors.

Overall, we did not find a compelling indication that the splicing aberrations present in mutants are linked to identifiable sequence features (Fig. 4I,J). The slight preference for stronger 5′ SS, BP site location (BPS) and intronic nucleotide composition are notable but will need further scrutiny using more sensitive experimental designs. However, pathway analysis indicated that DSE genes were significantly associated with mRNA processing (Fig. 4K). Thus, the relatively large number of splicing aberrations, compared to differentially expressed genes, detected at this developmental stage supports the hypothesis that these general splicing defects precede aberrations in gene expression and initiate the molecular cascade that leads to phenotypic changes.

### Increased skipping of *Mdm2* exon 3 and *Mdm4* exon 7, key regulators of P53, are associated with increased nuclear P53 in heads of *Snrpb^ncc+/−^* embryos

**Fig. 5.**
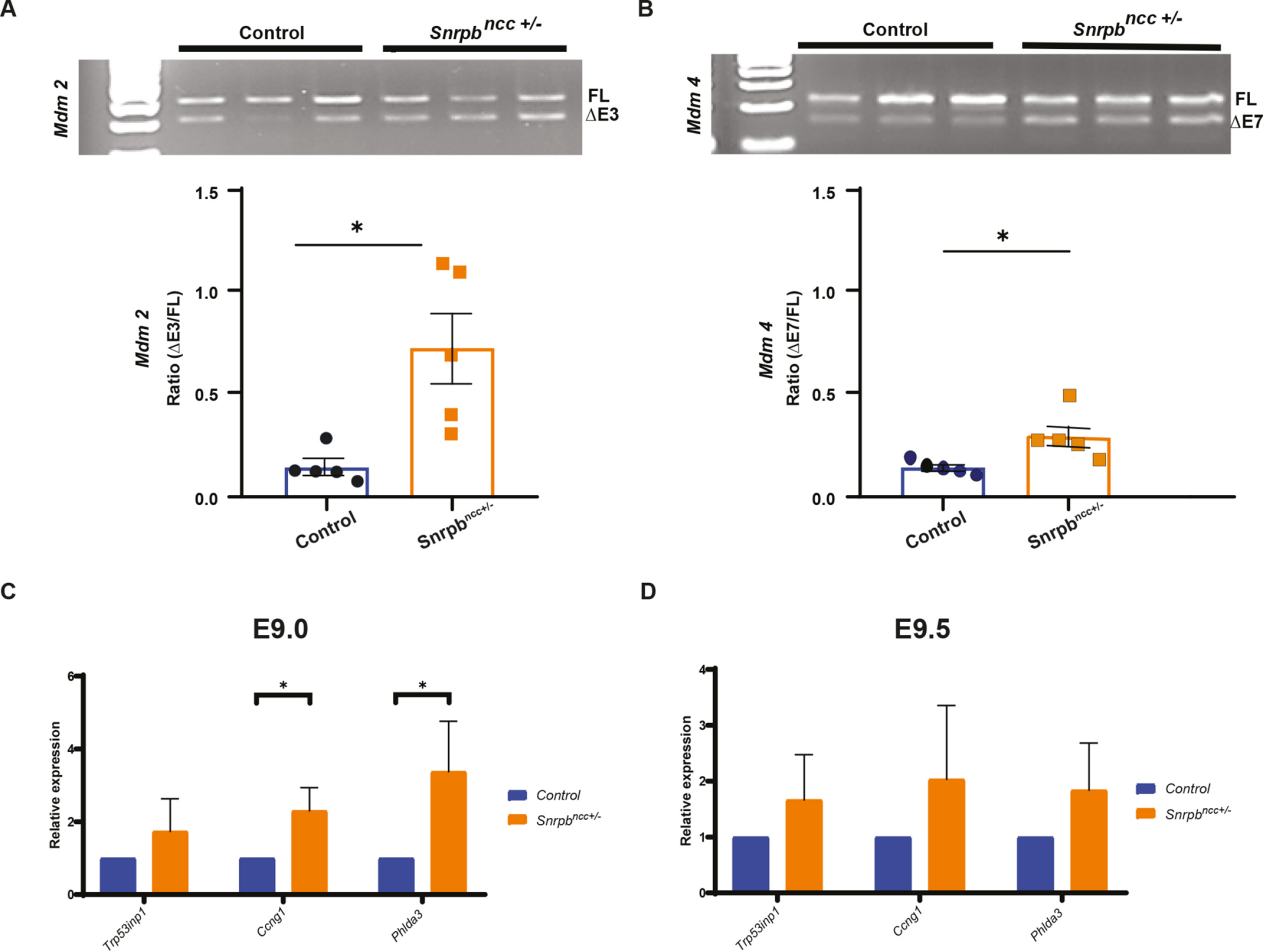
**Increased exon skipping in two regulators of P53, *Mdm2* and *Mdm4*, and significant increases in P53 target genes in *Snrpb^ncc+/−^* mutant heads.** (A,B) Representative images of RT-PCR showing long (FL) and short transcripts (ΔE) produced in *Mdm2* (A) and *Mdm4* (B) in E9.0 control (*Snrpb^wt^* or *Snrpb^L^*^/+^) and *Snrpb^ncc+/−^* embryos. Quantification revealed a significant increase in the ratio of *Mdm2* and *Mdm4* transcripts containing a skipped exon over the longer transcript in mutants (unpaired, two-tailed *t*-test, *P<0.05). Error bars indicate s.d. (C) Levels of P53 target genes were significantly increased in morphologically normal E9.0 *Snrpb^ncc^*^+/−^ embryos (*n*=5) compared to controls (*n*=5) (unpaired, two-tailed *t*-test, *P<0.05). (D) At E9.5, although levels of P53 target genes were increased in *Snrpb^ncc+/−^* embryos (*n*=3 group 1 and *n*=2 group 2), this difference was not significant. Error bars indicate s.e.m. FL, full length transcript; ΔE3, transcript with exon 3 skipped; ΔE7, transcript with exon 7 skipped.

We next investigated the key splicing changes that could explain craniofacial malformations in *Snrpb^ncc+/−^* embryos. We found increased skipping of exon 3 of *Mdm2* and exon 7 of *Mdm4*, regulators of the P53 pathway in our RNAseq analysis and confirmed these increases by reverse transcription PCR (RT-PCR) (Fig. 5A,B). Increased skipping of these exons was previously reported in cultured *Snrpb* knockdown cells and shown to increase levels of nuclear P53 in mouse embryos with mutations in *Eftud2*, a core component of the spliceosome (Beauchamp et al., 2021; Van Alstyne et al., 2018; Correa et al., 2016). Immunohistochemistry with an antibody against P53 revealed a significant enrichment of nuclear P53 in E9.5 mutant heads (Fig. S7A,B). Furthermore, levels of the P53-regulated genes *Trp53inp1*, *Ccng1* and *Phlda3* were increased, and this increase was statistically significant when levels of *Ccng1* and *Phlda3* were compared between E9.0 *Snrpb* WT and mutant embryos, but not at E9.5 (Fig. 5C,D). Thus, we conclude that the increased exon skipping in *Mdm2* and *Mdm4* results in increased nuclear P53 and levels of P53 target genes in *Snrpb^ncc+/−^* embryos, prior to morphological abnormalities. Because P53 activation can lead to increased apoptosis, we postulate that increased P53 activity contributes to apoptosis of *Snrpb^ncc+/−^* mutant cells.

### Reducing levels of P53 does not prevent craniofacial defects in *Snrpb^ncc/+^* embryos

We next tested whether reducing levels of P53 prevents craniofacial malformations in *Snrpb^ncc+/−^* embryos. We crossed *Trp53^loxP/+^*; *Wnt1-Cre2^tg^* mice and *Snrpb^loxP/+^* mice and collected E10.5 and E17.5 *Snrpb^ncc+/−^*; *Trp53^ncc^*^+/−^ double heterozygous embryos for analysis. We found no significant difference in the proportion of *Snrpb^ncc+/^*; *Trp53^ncc^*^+/−^ embryos with mild to severe craniofacial defects (*n*=4), compared to *Snrpb^ncc+/−^* mutants (*n*=3/3) (Fig. S7C,D; not shown). We then generated *Snrpb^ncc+/−^* with two mutant *Trp53* alleles in their neural crest cells (*Snrpb^ncc+/−^*; *Trp53^ncc^*^−/−^) for cartilage and skeletal analysis. E14.5 *Snrpb^ncc+/−^*; *Trp53^ncc^*^−/−^ mutant embryos (*n*=2) resembled *Snrpb^ncc+/−^* mutants found in group 2 (Fig. S7E). Similarly, E18.5 *Snrpb^ncc+/−^*; *Trp53^ncc^*^−/−^ mutant embryos (*n*=4) were morphologically similar to group 2 *Snrpb^ncc+/−^* mutants; they had microcephaly, a shorter snout and micrognathia (Fig. S7G). Cartilage and skeletal preparations revealed reduced ossification of the frontal bone, cleft palate, and asymmetric and abnormal development of the lower jaw (Fig. S7F,H-J). To determine whether homozygous deletion of *Trp53* improves or rescues survival of *Snrpb^ncc/+^* embryos, we allowed these mice to go to term and followed survival from P0 to P21, when the surviving pups were weaned. Of the 35 pups born, ten died within the first 2 days of life. Carcass was recovered for five of these dead pups, and genotyping revealed that they were all *Snrpb^ncc+/−^*; *Trp53^ncc^*^−/−^. None of the surviving pups (*n*= 25) were *Snrpb^ncc+/−^*; *Trp53^ncc^*^−/−^; chi-square analysis at P21 revealed this to be a significant deviation from expected Mendelian segregation (*P*=0.032). When we assumed that the dead pups were *Snrpb^ncc+/−^*; *Trp53^ncc^*^−/−^ embryos and performed a similar analysis, the significant difference was no longer found. Thus, we concluded that the other six pups that died between P1 and P2 were likely *Snrpb^ncc+/−^*; *Trp53^ncc^*^−/−^ mutants. In fact, *Snrpb^ncc+/−^*; *Trp53^ncc^*^−/−^ pups have not been found at P21 (*n*=0/36, four litters). Our data indicate that homozygous loss of *Trp53* alleviates the most severe defects associated with reduced levels of *Snrpb* and allows these mutant pups to survive to birth.

#### Abnormally spliced transcripts in *Snrpb^ncc+/−^* mutants include several required for craniofacial development

**Fig. 6.**
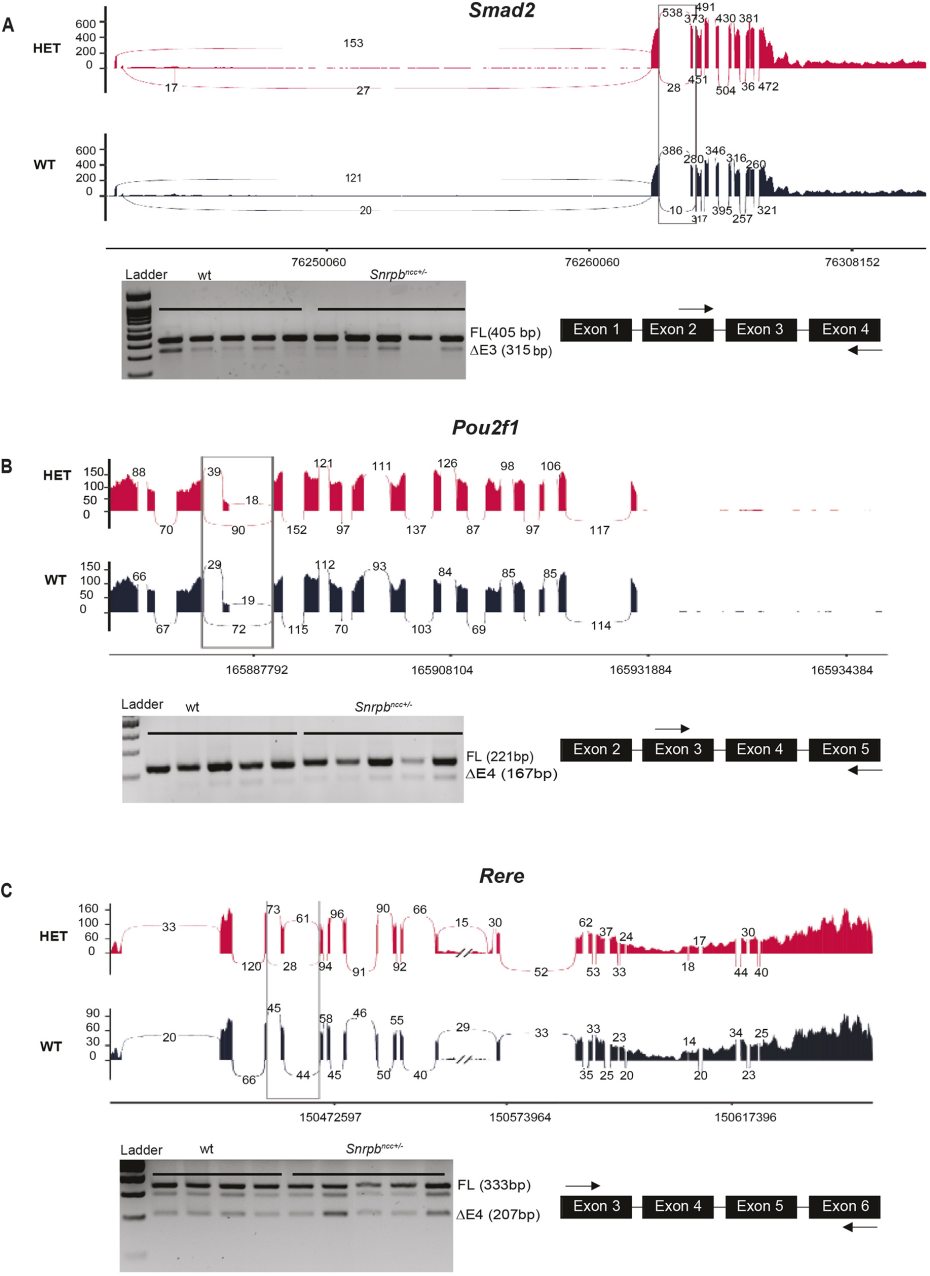
**Transcripts with exon skipping events are found in heads of E9.0 control and mutant embryos.** (A-C) Sashimi plots for the exon skipping events found for *Smad2* (A), *Pou2f1* (B) and *Rere* (C). Under each sashimi plot, representative gel for RT-PCR showing the presence of transcripts with the predicted exon skipping event. The location of primers used to amplify transcripts is shown on the right. No significant difference was found in the ratio of short/long transcripts (unpaired, two-tailed *t*-test). Error bars in the graphs indicate s.e.m. FL, full length; ΔE, skipped exon.

To identify additional abnormal splicing events that could explain craniofacial malformations in *Snrpb^ncc+/−^* embryos, we queried the Mouse Genome Informatics database to determine whether any transcripts with statistically significant DSEs were required for craniofacial development (Bogue et al., 2018). We identified 13 transcripts required for craniofacial development or stem cell development with significant increases in exon skipping (Table S1). Increased exon skipping in five of these genes – *Pdpk1*, *Rere* (*Atr2*), *Mcph1*, *Nf1* and *Dyrk2* – is predicted to introduce a pretermination codon. The remaining exon skipping events are not predicted to result in PTC but may alter gene expression and/or function. In fact, all except one of these DSEs were in constitutive exons. We then queried our RNAseq dataset to determine whether the expression level of these genes was altered in *Snrpb* mutants. We found no significant changes in levels of transcripts with PTC or non-PTC SEs. We then selected three transcripts and performed RT-PCR to confirm that the SE events identified in the RNAseq analysis were present in *Snrpb* mutant heads (Fig. 6). This analysis revealed the presence of transcripts with SEs for *Smad2*, *Pou2f1* and *Rere*, although the percentage of spliced events for *Smad2* and *Pou2f1* was below 10% and not significant when the ratio of short/long transcript in control and mutant was compared. We postulated that abnormal increases in exon skipping in these 13 transcripts, which are required for normal craniofacial development, may contribute to craniofacial defects in *Snrpb^ncc/+^* mutants.

### WT levels of *Snrpb* are required for normal expression of *Fgf8*, *Shh* and *Msx2*

**Fig. 7.**
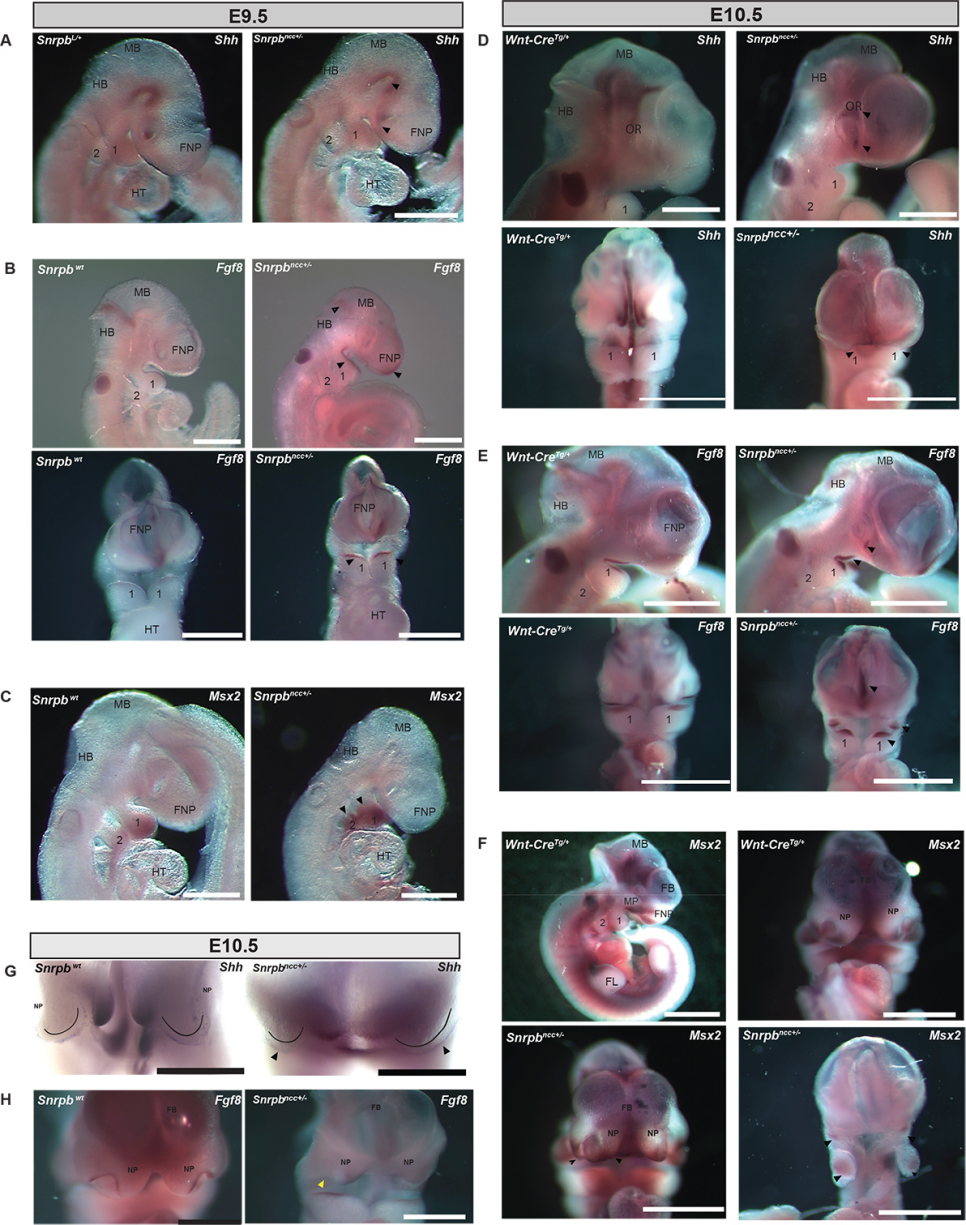
***Shh*, *Fgf8* and *Msx2* are mis-expressed in *Snrpb*^ncc+/−^ embryos.** Representative images of E9.5 and E10.5 control (*Wnt-Cre2^Tg/+^* or *Snrpb^wt^*) and *Snrpb^ncc^*^+/−^ embryos after wholemount *in situ* hybridization (ISH) to detect mRNA expression of *Shh*, *Fgf8* and *Msx2*. (A) *Shh* at E9.5 was expressed in the ventral-most region of the neural tube, the floor plate, and the ventral prosencephalon of control and *Snrpb^ncc+/−^* embryos (black arrowheads). (B) Lateral (top row) and ventral (bottom row) views of embryos showing that expression of *Fgf8* was expanded in the mandibular epithelium, the frontonasal prominence and the midbrain/hindbrain junction in group 2 (black arrowheads) (*n*=4) mutant embryos. (C) *Msx2* expression was extended proximally in pharyngeal arches 1 and 2 in group 1 mutant embryos (*n*=2; black arrowheads). (D) Wholemount ISH at E10.5. Top row: lateral view of embryos showing *Shh* expression in the diencephalon and ventral forebrain in a control (left) and group 2 mutant (right) embryo. Ectopic expression was found in the dorsal and ventral optic lens (arrowheads) of the mutant. Bottom row: ventral view showing expression of *Shh* on the oral ectoderm in a control (left) and group 2 mutant (right) embryo (black arrowheads). (E) Top row shows representative images of lateral views of a control embryo, in which *Fgf8* was detected on the surface ectoderm of the mandible, maxillary and frontonasal prominences (*n*=3). In the representative group 2 *Snrpb^ncc+/−^* mutant embryo, *Fgf8* expression was observed on the mandibular ectoderm and in the region where the maxillary prominence would normally form (black arrowheads; *n*=3); ectopic expression was also detected in the lens. Bottom row shows frontal view of reduced *Fgf8* expression in the hypoplastic lateral nasal prominence in mutants, and ectopic expression of *Fgf8* on the surface ectoderm of the medial nasal process, towards the midline (black arrowheads). (F) Top row shows a lateral (left) and ventral (right) view of *Msx2* expression in E10.5 control embryos. Bottom row shows that in E10.5 group 1 *Snrpb^ncc+/−^* mutants on the left, *Msx2* was expressed in the lateral and medial nasal prominences, although expression appeared reduced and ventrally expanded in the medial frontal nasal region. On the right, in a group 2 *Snrpb^ncc+/−^* mutant with a hypoplastic frontonasal and maxillary prominences, *Msx2* was expressed in the region where the maxillary prominences would form and in the mandibular region of the hypoplastic first arch. (G,H) Higher-magnification images of the facial ectoderm zone (FEZ) region of E10.5 embryos showing *Shh* expression in the developing mandibular periderm, and bilaterally on the surface ectoderm of the medial nasal prominences of control (G,H, left) and group 2 *Snrpb^ncc+/−^* (G,H, right) embryos (black arrowheads). (H) *Fgf8* expression in the lateral nasal prominence was reduced in group 2 mutants (yellow arrowhead), while ectopic expression of *Fgf8* was found on the surface ectoderm of the medial nasal process, towards the midline. 1, 2, pharyngeal arches 1 and 2; FNP, frontonasal process; HB, hindbrain; HT, heart; MB, midbrain; MP, mandibular process; NP, nasal process; OR, optic region. Scale bars: 500 μm.

The surface cephalic ectoderm, of which the facial ectoderm zone (FEZ) is a subregion, is essential for integrating proper growth of the craniofacial skeletons and brain and patterning the underlying neural crest (Griffin et al., 2013). The severe malformations found in the face and brain of *Snrpb* mutants suggest that the FEZ might not have formed. Therefore, we used *in situ* hybridization to examine expression of *Shh* and *Fgf8*, which are expressed in surface cephalic ectoderm and together help to define the FEZ (Griffin et al., 2013). At E9.5, before the FEZ forms, *Shh* was expressed in the ventral-most region of the neural tube, the floor plate, and the ventral prosencephalon of *Snrpb* WT and *Snrpb^ncc+/−^* embryos (*n*=4; *n*=3 group 1 and *n*=1 group 2) (Fig. 7A). At this stage, *Fgf8* was expressed in the mandibular epithelium, the frontonasal prominence, and the midbrain/hindbrain junction of control and *Snrpb^ncc+/−^* mutant embryos. However, the expression domain of *Fgf8* was abnormally expanded at these sites (*n*=4; *n*=2 group 2 and *n*=2 group 1) (Fig. 7B). Furthermore, expression of *Msx2*, a downstream target of *Fgf8* in the underlying neural crest cells (Griffin et al., 2013), was also abnormal in E9.5 *Snrpb^ncc+/−^* embryos. In *Snrpb^ncc+/+^* embryos (*n*=6), *Msx2* was expressed in the distal region of pharyngeal arches 1 and 2 (Fig. 7C). However, in *Snrpb^ncc+/−^* embryos, *Msx2* expression was abnormally extended proximally in these arches (*n*=4; *n*=3 group1 and *n*=1 group 2) (Fig. 7C). These *in situ*s revealed that normal levels of *Snrpb* are required in the neural crest cells to restrict expression of *Fgf8* and its downstream target *Msx2* in the developing head and face in both morphologically normal and abnormal embryos.

In E10.5 group 2 *Snrpb^ncc+/−^* mutant embryos missing the frontonasal prominence, expression of *Shh* was found in the diencephalon and the ventral forebrain (*n*=3; Fig. 7D). Furthermore, ectopic *Shh* expression was found in the dorsal and ventral optic lens (arrowheads in Fig. 7D). In group 1 mutant embryos, the lateral and medial nasal processes were further apart than in WT embryos, but *Shh* expression was found in the developing mandibular periderm and bilaterally on the surface ectoderm of the medial nasal prominences (arrowheads in Fig. 7G; *n*=1). In E10.5 WT embryos, *Fgf8* was expressed on the surface ectoderm of the mandible, the maxillary prominences and frontonasal prominences. In group 2 *Snrpb^ncc+/−^* mutant embryos with hypoplastic frontonasal and maxillary prominences, expression of *Fgf8* was found on the mandibular ectoderm and in the region where the maxillary prominence would normally form (*n*=2; Fig. 7E). In group 1 E10.5 *Snrpb^ncc+/−^* embryos, *Fgf8* expression in the lateral nasal prominence was reduced, while ectopic expression of *Fgf8* was found on the surface ectoderm of the medial nasal process, towards the midline (*n*=3; Fig. 7H). Similarly, in E10.5 group 2 *Snrpb^ncc+/−^* mutants missing the frontonasal and maxillary prominences, *Msx2* expression was expressed in the maxillary and in the mandibular region of the hypoplastic first arch (*n*=3). In group 1 *Snrpb^ncc+/−^* mutants, *Msx2* was expressed in the lateral and medial nasal prominences, although expression appeared reduced but ventrally expanded in the medial frontal nasal region (*n*=2; Fig. 7F). Thus, in *Snrpb^ncc+/−^* embryos in which the lateral and medial nasal prominences formed, reduced expression of *Fgf8* in the ectoderm results in abnormal expression of *Msx2* in the underlying neural crest. We postulate that DSEs in genes important for midface development lead to abnormal expression of *Shh*, *Fgf8* and *Msx2* expression and mis-patterning of the developing craniofacial region.

## DISCUSSION

Splicing is an essential and ubiquitous process that generates mature mRNAs and increases the number and diversity of proteins from the genome (Chen and Manley, 2009; Nilsen and Graveley, 2010). SNRPB is an essential protein that facilitates assembly of the snRNP proteins that carry out splicing. Surprisingly, mutations that increase levels of a non-functional *SNRPB* transcript result in CCMS, a craniofacial spliceosomopathy that is also associated with rib defects (Lynch et al., 2014; Bacrot et al., 2015; Beauchamp et al., 2020). To examine the role of *SNRPB* in the development of tissues affected in patients, we first designed guide RNAs (gRNAs) and repair templates to generate a mutation in alternative exon 2 that would model those found in CCMS patients. However, this strategy did not prove to be fruitful as we did not recover mutant mouse lines carrying this mutation. Therefore, we generated a conditional mutant mouse line to test whether WT levels of *Snrpb* were required for normal development. Herein, we showed that *Snrpb* is haploinsufficient in mice and required for normal splicing of key regulators of P53 and transcripts required for normal craniofacial development, as well as expression of *Fgf8* and *Shh*. We show that morphological defects in *Snrpb* mutants were not associated with significant changes in gene expression but with disruptions in alternative splicing and patterning of the craniofacial region. We suggest that altered transcript ratios and expression of genes important for patterning the craniofacial region are responsible for malformations and embryonic death of mutant embryos.

In our conditional mutant mouse line, deletion of exon 2, alternative exon 2 and exon 3 in the presence of Cre is predicted to generate a shorter *Snrpb* transcript of 527 bp that encodes for a non-functional protein. When *β-actin*-*Cre* was used to delete the *loxP*-flanked exons, the resulting *Snrpb* heterozygous embryos died post-implantation*.* Therefore, we were unable to study the role of SNRPB in head and rib development in these mutants. Although further studies are needed to determine whether there is a general growth defect or other roles for *Snrpb* at these early stages of development, our study indicates that heterozygosity for a loss-of-function allele of *Snrpb* is lethal. In fact, a single patient carrying a mutation in the 5′ UTR of *SNRPB* that was predicted to result in a null allele was more severely affected and failed to survive gestation (Lynch et al., 2014). Thus, our data support the hypothesis that *SNRPB* mutations commonly found in CCMS patients are not null mutations (Lynch et al., 2014).

To study the role of SNRPB in craniofacial development, we used the *Wnt1-Cre2* transgenic mouse line to generate embryos with heterozygous mutation of *Snrpb* in their neural tube and neural crest cells. In *Snrpb^ncc+/−^* mutant embryos, craniofacial structures derived from neural crest cells, such as the nasal bone, palates, maxilla, mandible and middle ear structures, are abnormally formed. Intriguingly, these structures are also commonly reported to be affected in CCMS patients, suggesting that abnormal neural crest cell survival and/or differentiation are responsible for defects in those patients. We also uncovered absence or reduction of the hyoid bone and ectopic cartilage and bones that we could not identify in some *Snrpb^ncc+/−^* mutants. Similarly, accessory ossicles in the hyoid bone were found after CT scan of two CCMS patients by Tooley et al. (2016). We postulate that SNRPB is required in all neural crest cells and their derivatives, a hypothesis that is supported by the reduced or absent aorticopulmonary septum that is found in the micro-CT scan of *Snrpb^ncc+/−^* mutants. Furthermore, we propose that aorticopulmonary septal defects and or palatal clefts such as the ones found in E17.5 mutants contribute to death of *Snrpb^ncc+/−^* embryos, as was found in *Eftud2^ncc−/−^* mutants (Beauchamp et al., 2021). Finally, although phenotypes found in CCMS patients strongly suggested a requirement of SNRPB for endochondral ossification, our data show abnormal development of bones formed via both endochondral and intramembranous ossification, indicating an early role for SNRPB in skeletal development.

P53 stability and activity are known to be upregulated in response to mutation or disruption in the level of splicing factors (Van Alstyne et al., 2018; Correa et al., 2016, Lei et al., 2017; Zhu et al., 2020). In fact, we found increased skipping in two P53 regulators, *Mdm2* exon 3 and *Mdm4* exon 7, increased nuclear P53 and upregulation of P53 target genes in heads of E9.0 *Snrpb^ncc+/−^* mutants. In zebrafish and mouse, increased P53 activity contributes to craniofacial defects, and knocking down or removing P53 genetically reduced apoptosis and improved development (Lei et al., 2017; Mao et al., 2016; Jones et al., 2008). Additionally, we showed that the P53 inhibitor, Pifithrin-α, improved head and brain development in embryos with mutation of *Eftud2* in the neural tube and neural crest (Beauchamp et al., 2021). However, reducing or removing P53 genetically in the neural crest cells did not prevent craniofacial defects in *Snrpb^ncc+/−^* mutant embryos. Although the variable expressivity found in *Snrpb^ncc+/−^* embryos makes it difficult to rule out a partial rescue, our findings indicate that P53 alone is probably not responsible for the malformations that we found.

RNAseq is a sensitive method for examining gene expression (Wang et al., 2009), and our data indicate that reduced expression of SNRPB in mutant neural crest disrupts splicing and expression of genes important for craniofacial development. In fact, our RNAseq analysis using the head of morphologically normal E9.0 *Snrpb^ncc+/−^* embryos revealed many more DSEs than DEGs. We confirmed differential splicing of the P53 regulators *Mdm2* and *Mdm4*, which may lead to increased apoptosis, and identified 13 transcripts important for craniofacial development that were abnormally spliced in *Snrpb^ncc+/−^* embryos. A DSE may perturb gene expression levels, for example with the introduction of pretermination codon, or alter the activity or localization of the resulting gene product. For example, the DSE associated with exon 3 of *Smad2* is predicted to increase the proportion of transcript that encodes a much more potent effector of TGFβ/Nodal than the full-length SMAD2. This shorter protein heterodimerizes with SMAD3 to regulate many developmental processes, including growth of the mandible (Dunn et al., 2005). Similarly, increased skipping of exon 8 of *Ror2* may disrupt the ability of this receptor to interact with *Wnt5* during midface, ear and jaw development (Schwabe et al., 2004). Furthermore, deletions of constitutive exons may change the open reading frame, insert a pretermination codon or generate an unstable transcript that is removed by nonsense-mediated decay. Hence, we predict that an increase in the proportion of transcripts with a missing constitutive exon will reduce levels of the associated proteins. Abnormal expression of *Fgf8*, which is regulated by SMAD2 (Liu et al., 2004), and *Rere* (Kumar and Duester, 2014) may be one of the consequences of mis-splicing. Nonetheless, reduced migration of neural crest cells into the frontonasal region could also explain abnormal expression of *Fgf8* and *Shh.* Although we found no significant differences in the number of neural crest cells in heads of E9.0 control and *Snrpb* mutant embryos, reduced levels of *Nisch*, which binds to integrins to block cell migration (Ding et al., 2008), may disrupt migration of a specific subset of neural crest cells that cannot be identified with our current techniques. In the future, we will investigate the contribution of DSEs to abnormal expression of *Fgf8* and *Shh* and to craniofacial defects in *Snrpb* mutants.

If, as we postulate, malformations in *Snrpb* mutants are due to a DSE that leads to increased cell death and disruption of multiple pathways important for patterning, the variable penetrance found in mutants may reflect the proportion of cells that undergo cell death and the level of disruption in patterning. Thus, embryos in which a large number of cells die would have absent craniofacial structure formations, and resemble group 3 or 4, whereas a lesser amount of cell death would lead to mutants classified as group 1/2. Furthermore, for those in group 1 and 2, the severity of craniofacial malformation would then depend on the level of DSEs in the genes critical for patterning of the region. The absence of group 3 or 4 *Snrpb; Trp53* mutants supports this hypothesis. Loss of P53 may reduce cell death and allow for development of craniofacial structures in these mutants. However, DSEs in patterning genes are presumably independent of P53 and may lead to malformations and embryonic death. Future characterization of cell death and patterning in *Snrpb; Trp53* double mutant embryos, along with RNAseq experiments using morphologically normal and abnormal mutant heads, may allow us to tease out these different contributors to craniofacial malformation and aid in identifying DSEs and pathways regulated by Snrpb.

Our working model is that dysregulation in the level of SNRPB, even if modest – as is likely the case with inclusion of the PTC-containing alternative exon 2, perturbs the efficiency of splicing at the level of spliceosome assembly. Furthermore, although cells with reduced levels of *Snrpb* have an increased propensity to undergo apoptosis, increased DSEs are found before they die. Therefore, we propose that splicing changes in important developmental genes, the proportion of cells that undergo apoptosis, and the timing of apoptosis may all contribute to the variable expressivity found in *Snrpb* heterozygous mice and in CCMS patients. In conclusion, we believe that our work using the first CCMS animal model shows evidence for both ubiquitous and development-specific roles of *Snrpb* during morphogenesis and provides much needed insights into the role of this splicing factor during embryogenesis.

## MATERIALS AND METHODS

### Materials

All antibodies, chemicals and most mouse lines used in this study are commercially available. All other unique materials are available upon request.

### Mouse lines

All procedures and experiments were performed according to the guidelines of the Canadian Council on Animal Care and approved by the animal Care Committee of the Montreal Children's Hospital. WT CD1, mT/mG *Gt(ROSA)26Sor^tm4(ACTB-tdTomato,-EGFP)Luo^/J*, (Soriano, 1999), *Wnt1-Cre2* (Lewis et al., 2013) and *β-actin-Cre* (Lewandoski et al., 1997) mice on the C57BL/6J genetic background were purchased from The Jackson Laboratory. The *R26R* strain [*Gt (ROSA)26Sor^tm1Sor^* (Muzumdar et al., 2007)] on the mix C57BL/6J;129/S4 genetic background was a kind gift from Dr Nagano (Department of Obstetrics and Gynecology, McGill University, Montreal, Canada). All strains were maintained on the CD1 genetic background. The *Trp53^tm1brn^* mouse line with *loxP* sites flanking exons 2-10 of the *Trp53* gene was purchased from The Jackson Laboratory (*Trp53^loxP/+^*) (stock #008462) (Marino et al., 2000).

### Generation and establishment of *Snrpb* conditional mutant mouse lines

To develop a conditional knockout *Snrpb* allele, we used a CRISPR/Cas9-mediated homology-directed repair strategy to insert *loxP* sequences in intron 1 and intron 3 to flank exons 2 and 3. Candidate efficient gRNAs were selected based on previous references (Xu et al., 2015). Microinjection with single-strand DNA template, gRNAs and Cas9 mRNA was performed. In the first round of microinjection targeting intron 1, a *loxP* sequence was inserted in intron 1 of 25% of the animals born. The insertion was confirmed in two animals (one male and one female) by Sanger sequencing. We generated homozygous animals with *loxP* sequences in intron 1 from those mice. Intron 1 homozygous *loxP* animals were then used for the second round of microinjection to insert *loxP* in intron 3. Sanger sequencing of DNA from a G1 male offspring of a targeted founder from the second round of microinjection and a WT CD1 female was used to confirm that both *loxP* sequences in intron 1 and intron 3 were intact. Thereafter, we backcrossed the animals for at least five generations to establish the *Snrpb* conditional mutant mouse line and to remove any potential off-target effect from CRISPR editing. All embryo analysis was of embryos on the mix CD1; C57BL/6J genetic background.

### Generation of *Snrpb^+/−^* mutant embryos

*Snrpb^+/−^* mutants were generated by crossing *Snrpb^loxP/+^* mice with *β-actin-Cre^Ttg/+^* mice.

### Generation neural crest cell-specific *Snrpb^+/−^* mutants

To generate embryos and animals with neural crest-specific *Snrpb* heterozygosity, *Wnt1-Cre ^Tg/+^* animals were mated with *Snrpb^loxP/+^* mice. Embryos obtained from these mating were *Snrpb* heterozygous mutant in the neural crest cells and their derivatives; all others were *Snrpb* WT.

### Genotyping of mice and embryos

Genomic DNA was extracted from mouse tails or yolk sacs by alkaline lysis (Hou et al., 2017). For *Snrpb*, genotyping was performed to identify the WT and conditional allele (with *loxP* sequences) to amplify segments of intron 1 using the following program: 30 s 95°C, 30 s 62°C, 30 s 72°C for 35 cycles followed by an elongation step of 10 min at 72°C. This PCR amplified the targeted DNA segment to determine a WT (347 bp) and a mutant (387 bp) amplicon. The primers used for the genotyping were as follows: forward, 5′-CCCGAGACAGACACAACATAAG-3′; reverse, 5′-GCTTTGAAGGTCCCGATGAA-3′. For the commercially available lines, namely R26R, *Wnt1-Cre2*, mT/mG and *β-actin-Cre*, genotyping was performed as detailed on The Jackson Laboratory website: protocol #29915 (*R26R*), #25394 (*Wnt1-Cre2*), #20368 (mT/mG) and #33618 (*β-actin-Cre*), respectively.

### Collection of embryos

The day of plug was considered E0.5. On the day of dissection, embryos were dissected from their extraembryonic membranes. Yolk sacs were used for genomic DNA extraction and genotyping. All embryos were assessed for the presence of a heartbeat, and somite number was counted between E8.5 and E10.5. Embryos were fixed in 4% paraformaldehyde in PBS at 4°C overnight (unless otherwise stated), washed and kept in PBS at 4°C until use.

### Wholemount *in situ* hybridization and preparation of embryos for embedding

Fixed embryos were dehydrated using a graded methanol series for wholemounts. Wholemount RNA *in situ* hybridization was performed as previously described (Revil and Jerome-Majewska, 2013). For cryo-embedding, fixed embryos were first cryoprotected in 30% sucrose overnight, embedded in Cryomatrix and stored at −80°C until sectioning.

### Cartilage staining of embryos and skeletal preparation of embryos and pups

To investigate cartilage formation, embryos were stained with Alcian Blue. For skeletal staining, the skin was removed from freshly dissected E17.5 embryos and neonatal pups and stained as described by Wallin et al. (1994). We measured the mandible length from the incisor to the articular surface of the condyloid process. In mutants in which the processes of the mandibles were not properly formed, the incisor to the proximal end of the mandible was measured.

### Wholemount X-gal staining of embryos

Embryos were stained with freshly prepared X-gal staining solution overnight at 37°C in the dark as described in Beauchamp et al. (2021). Post-staining, embryos were embedded in Cryomatrix and stored at −80°C until sectioning. ImageJ was used to quantify the X-gal-stained area.

### Phosphotungstic acid staining for CT

Embryos were fixed overnight in 4% paraformaldehyde and then washed with PBS. After a series of dehydration steps according to the protocol described previously (Lesciotto et al., 2020), embryos were stained in 0.7% phosphotungstic acid (PTA). The duration of staining varied depending on the stage of the embryos, and pre-scanning was done to confirm complete penetration of the PTA. Once all the structures were visualized in the pre-scan, embryos were rehydrated in a series of methanol and CT scanning was done at 20-µm thickness.

### Immunofluorescence and TUNEL assay

Immunofluorescence experiments were performed on 10-μm-thick sections according to standard protocols (Zakariyah et al., 2012). Primary antibody used was anti-PH3 (Ser10) (06-570, Millipore; 1:200 dilution). Alexa Fluor 568 (A-11004, ThermoFisher Scientific; 1:500 dilution) secondary antibody was used. For identifying cells undergoing apoptosis, TUNEL assay using a Cell Death Detection Kit, TMR Red was used (12156792910, Roche). For quantification of fluorescence signal, particle analysis on ImageJ was used. For TUNEL and PH3 quantification, at least four sections were counted per embryo and per genotype.

### Immunohistochemistry

Embryos were sectioned at 10-μm thickness for immunohistochemistry as previously described (Beauchamp et al., 2021; Hou et al., 2017). Anti-P53 primary antibody (2524, Cell Signaling Technology; 1:250 dilution) or anti-neurofilament primary antibody (2H3, Developmental Studies Hybridoma Bank; 1:150 dilution) was used. A VECTASTAIN^®^ Universal Quick HRP Kit was used as secondary antibody and visualized with diaminobenzidine (DAB).

### RNA isolation for RNAseq

RNA extraction was done using a Qiagen RNeasy kit, following the manufacturer's protocol, from samples stored in RNAlater (Invitrogen). For RNA isolation at E9.0, heads of two somite-matched embryos from different litters were pooled according to genotype. Three WT and heterozygous pools were used for RNAseq analysis.

### Quantitative RT-PCR

Total RNA was treated with DNAse (NEB; according to the manufacturer's protocol) and used for reverse transcription with iScript™ Reverse Transcription Supermix for RT. Quantitative RT-PCR was performed using Advanced Universal SYBR^®^ Green Supermix. Experiments were performed in duplicates to ensure technical replicability. Target genes were normalized with the normalization factor as calculated by geNorm software (Vandesompele et al., 2002). Three housekeeping genes – *B2m*, *Gapdh* and *Sdha* – were used for generation of the normalization factor as previously reported (Vandesompele et al., 2002).

### RNAseq analysis

Sequencing libraries were prepared by the McGill Genome Centre (Montreal, Canada), using a TruSeq Stranded Total RNA Sample Preparation Kit (TS-122-2301, Illumina, San Diego, CA, USA) by depleting ribosomal and fragmented RNA, synthesizing first- and second-strand complementary DNA (cDNA), adenylating the 3′ ends and ligating adaptors, and enriching the adaptor-containing cDNA strands by PCR. The libraries were sequenced using an Illumina NovaSeq 6000 PE100 sequencer, with 100 nucleotide paired-end reads, generating between 109 and 230 million reads sample. The sequencing reads were trimmed using CutAdapt (Martin, 2011) and mapped to the mouse reference genome (mm10) using STAR (Dobin et al., 2013) aligner (version 2.6.1d), with default parameters, and annotated using the Gencode (Harrow et al., 2006) M2 (version M2, 2013) annotation. Htseq-count [part of the ‘HTSeq’ (Anders et al., 2015) framework, version 0.13.5] was used for expression quantification.

To perform differential splicing analysis, rMATS 4.0.2 (Shen et al., 2014) was used, and detected splicing events were filtered by systematically excluding those with a mean of inclusion junction counts lower than 5 in either WT or heterozygous samples. To identify a significant DSE, an absolute inclusion level difference cut-off of more than 0.05 was used and a Benjamin–Hochberg multiple testing correction with an FDR cut-off of less than 0.1 was used. The rationale for relaxing the FDR cut-off here was to obtain a large dataset enriched for alternative splicing events in order to observe general tendencies, such as increased propensity for exon skipping or intron retention in the mutants. To characterize 3′ SS sequences, LaBranchoR (Paggi and Bejerano, 2018), a BP prediction tool based on a deep-learning approach was used, which uses a bidirectional long short-term memory network model to identify relevant BPs upstream of DSEs. The BPs and their surrounding area consensus motifs were generated using WebLogo 3.0 (Crooks et al., 2004).

For differential expression analysis (DEA), we used DESeq2 (Love et al., 2014) package, and a list of significant DEGs was derived using an FDR cut-off of less than 0.05 with no additional restriction on the absolute log2 fold change (Log2FC) (to allow for detection of even minor expression changes). For Kyoto Encyclopedia of Genes and Genomes (KEGG) pathway analyses, the combined list of up- and down-regulated genes from DEA was used as input to gProfiler2 (Raudvere et al., 2019) package (gost function), and all the detected genes from DEA were used as background.

A differential analysis of transposable element (TE) and long non-coding RNA (lncRNA) expression was also carried out, to investigate whether SNRPB deficiency may result in deregulation of the non-coding transcriptome. Those analyses did not uncover any differences in the mutant embryos, and the results are not shown in the paper. STAR was used to map the processed reads with modified options: —outFilterMultimapNmax 100 —winAnchorMultimap Nmax 100 —outMultimapperOrder Random —alignSJoverhangMin 8 —outFilterMismatchNmax 999 —alignIntronMin 20 —alignIntronMax 1000000 —alignMatesGapMax 1000000, with mouse annotations to guide mapping, coming from the University of California, Santa Cruz RepeatMasker (Gencode M1) and lncRNA (Gencode M1) annotations. The mapped lncRNA and TE reads were respectively quantified with salmon (Patro et al., 2017) and TElocal (Jin et al., 2015). Differential lncRNA and TE expression analyses were performed using DESeq2, with the TE and lncRNA read counts being normalized using protein-coding gene expression size factors, and differentially expressed lncRNA and TEs selected based on an FDR cut-off of less than 0.05 and an absolute Log2FC of greater than 0.5 to increase detection signal.

### Quantification and statistical analysis

Quantitation was performed using ImageJ software (National Institutes of Health, Bethesda, MD, USA). Statistical analyses were conducted using Prism 8.0 software (GraphPad, San Diego, CA, USA). Chi-square test or non-parametric Mann–Whitney U-test analysis was performed using Prism. *P*<0.05 was considered significant.

## Supplementary Material

Supplementary information
