# Supplementary material for: Snrpb is required in murine neural crest cells for proper splicing and craniofacial morphogenesis
Source: Dis Model Mech. 2022 Jun 23;15(6):dmm049544. doi: 10.1242/dmm.049544 (PMC9235875; doi:10.1242/dmm.049544)
Supplement: Supplementary information [file dmm-15-049544-s1.pdf]

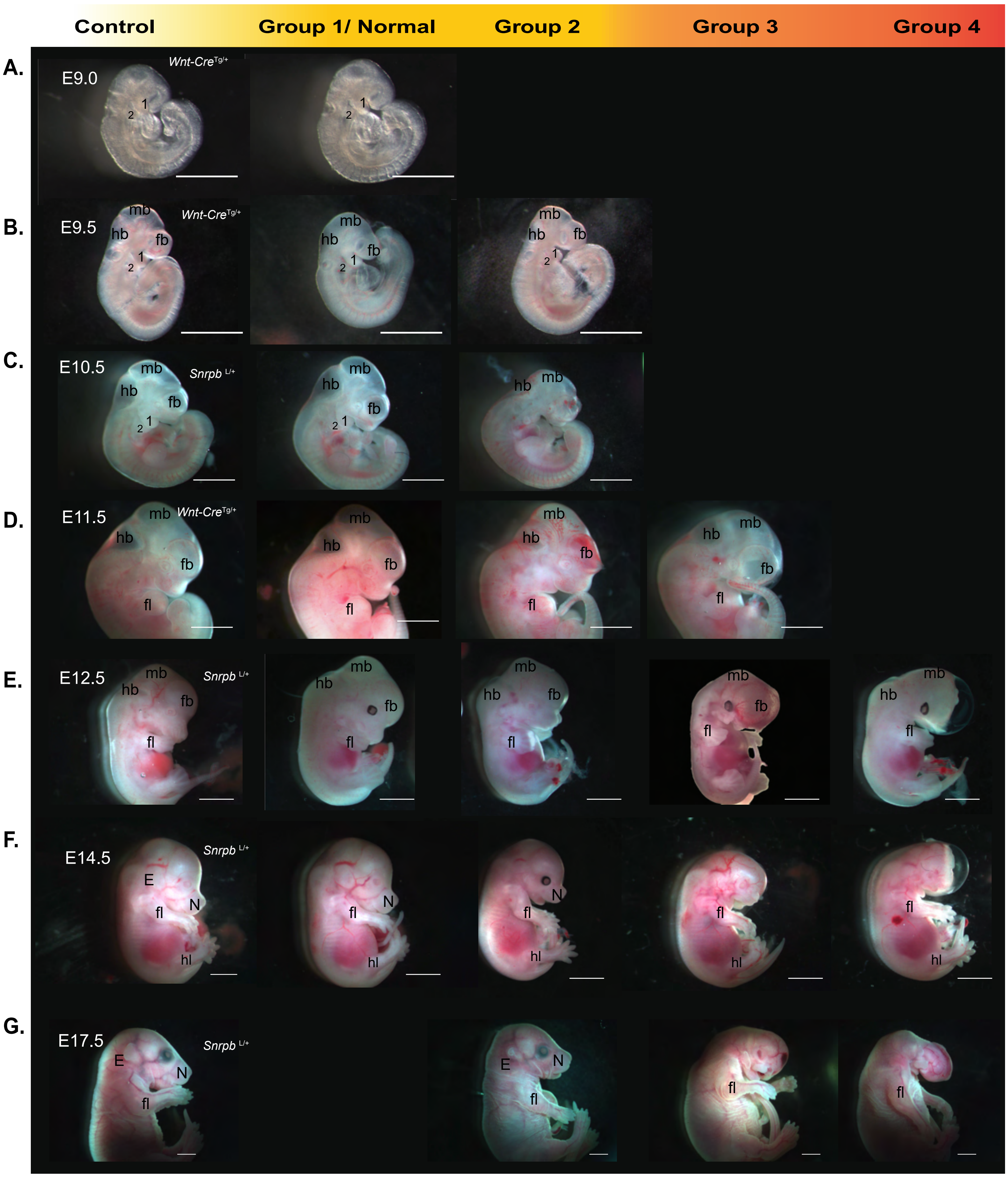

Disease Models & Mechanisms • Supplementary information

Fig. S1. *Snrpb*$^{ncc+/-}$ embryos show craniofacial malformations of varying expressivity from E9.5 onward. Representative images showing control (*Wnt1-Cre*$^{Tg/+}$ or *Snrpb*$^{L/+}$) and *Snrpb*$^{ncc+/-}$(*Snrpb*$^{L/+}$; *Wnt1-Cre*$^{Tg/+}$) embryos grouped by age and phenotype **A**. At E9.0, all *Snrpb*$^{ncc+/-}$embryos were morphologically normal and resembled control littermates, they were classified as group 1. **B**. At E9.5, 50% of *Snrpb*$^{ncc+/-}$ embryos were morphologically normal and 50% had hypoplastic forebrain, midbrain and hindbrain and were classified as group 2. **C**. Morphologically normal E10.5, *Snrpb*$^{ncc+/-}$ embryos were assigned to group 1, and abnormal embryos that showed reduced frontonasal prominence, small pharyngeal arches, forebrain and midbrain were classified as group 2. **D**. E11.5 *Snrpb*$^{ncc+/-}$ mutants were classified into three groups. Group 1 was morphologically normal; group 2 had hypoplasia of the forebrain, midbrain and hindbrain as wells as reduced maxillary and mandibular prominences; group 3 mutants had severe hypoplasia of the frontonasal, maxillary prominences and the mandibular prominence. **E-G**. At E12.5 (**E**), E14.5 (**F**) and E17.5 (**G**), *Snrpb*$^{ncc+/-}$ mutants were classified into 4 groups. Group 1 was morphologically normal; group 2 had frontonasal and mandibular clefts; group3 had reduced and abnormal forebrains with clefts in their hypoplastic frontonasal and mandibular prominences; and group 4 mutants did not have a morphologically identifiable forebrain or anterior facial structures. **G**. Group 1 or morphologically normal embryos were not found at E17.5. 1, 2= pharyngeal arch 1 and 2, respectively, fb=forebrain, mb=midbrain, hb=hindbrain, fl=forelimb, E=ear, N=nose, hl=hindlimb.

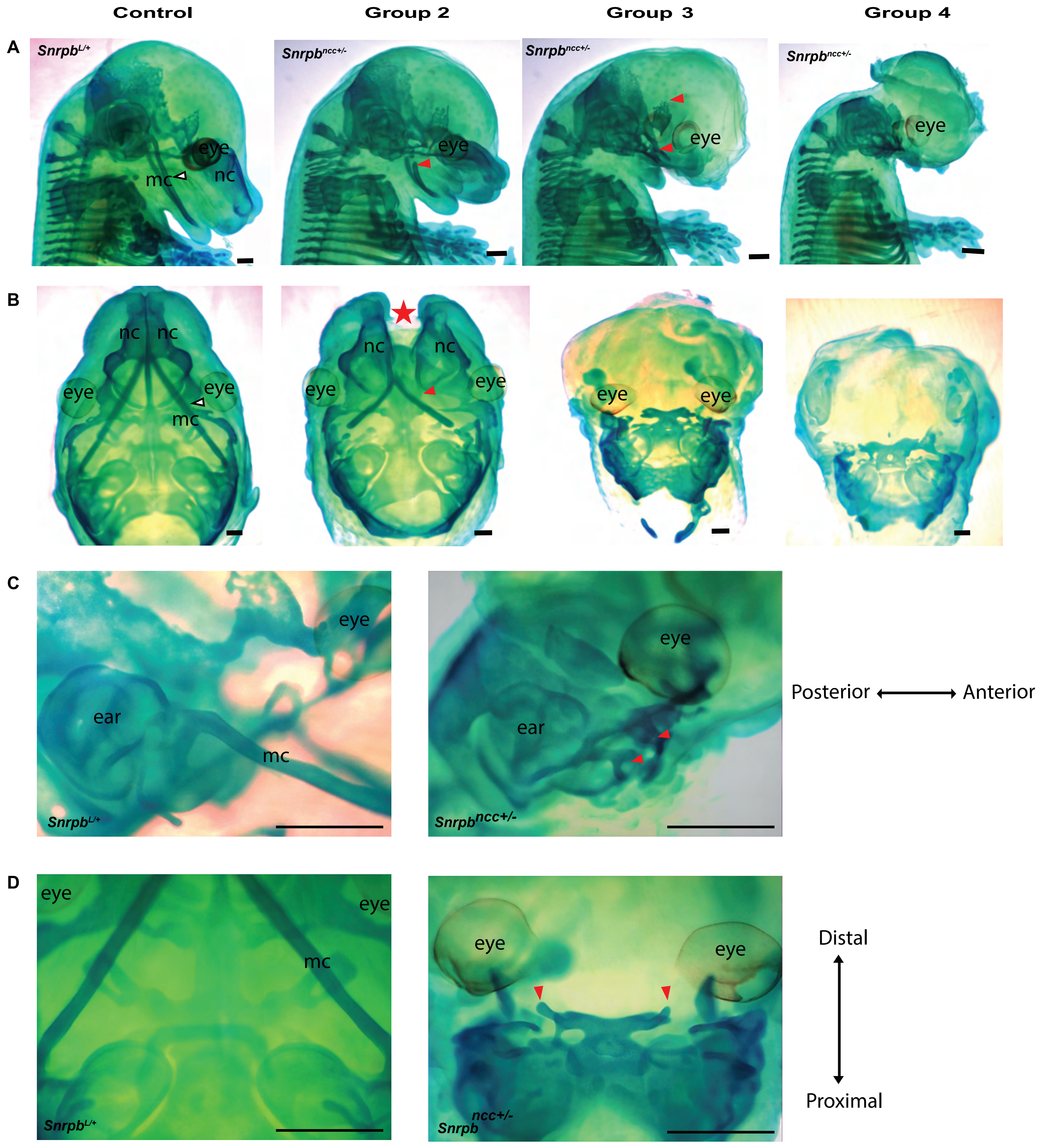

Fig. S2. E14.5 *Snrpb*$^{ncc+/-}$ mutants show hypoplastic and loss of craniofacial cartilage development. Representative images of E14.5 control (*Snrpb*$^{L/+}$) and *Snrpb*$^{ncc+/-}$ embryos stained with Alcian blue **A**. Representative images showing sagittal views of a wild type, control, embryo with normal development of head, nasal and Meckel's cartilage (white arrowhead); a group 2 *Snrpb*$^{ncc+/-}$ group 2 mutant with a shorter and discontinuous Meckel's cartilage (mc) (red arrowhead); a group 3 mutant with reduced head cartilage (red arrowhead), ectopic cartilage in the maxillary prominence (red arrowhead), and an absent nasal cartilage; a group 4 mutant with absent anterior craniofacial cartilages **B**. Representative images showing ventral views of the head of a wildtype, control, embryo with symmetrical Meckel's cartilage (white arrowhead) and nasal cartilage; in the group 2 mutant Meckel's cartilage is truncated and asymmetrical (red arrowhead) and the nasal cartilage is clefted (red star); in group 3 and 4 mutants, ventral cartilages are missing. **C**. and **D**. Higher magnification of the ear (lateral view) and cranial base (ventral view) of control and a group 3 *Snrpb*$^{ncc\ +/-}$ mutant indicating ectopic cartilages found in a subset of mutants (n=4/7), red arrowheads. mc=Meckel's cartilage, nc=nasal cartilage.

Disease Models & Mechanisms • Supplementary information

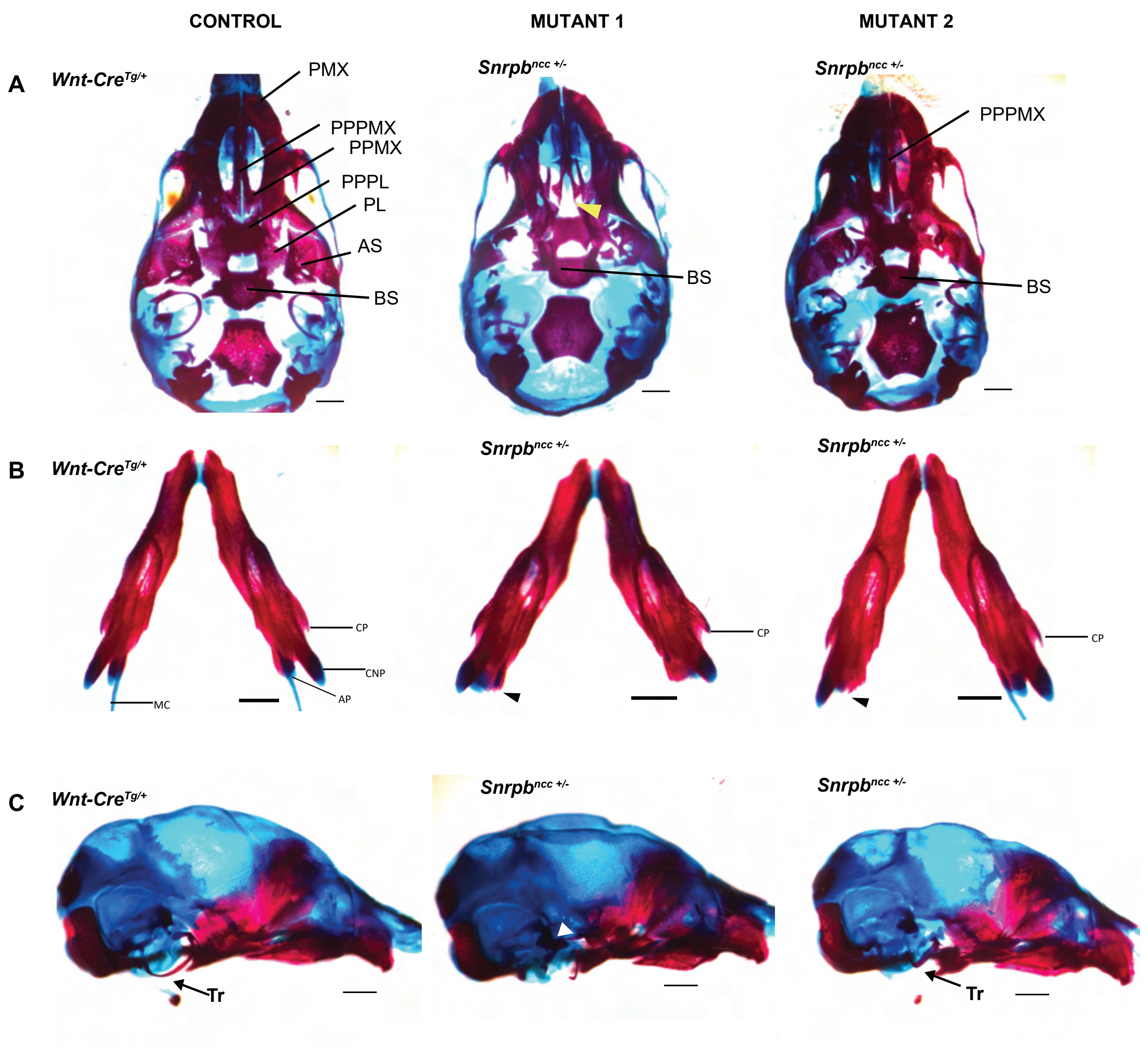

Fig. S3. Craniofacial malformations in newborn *Snrpb*$^{ncc+/-}$ mutants. Representative images of P0 control (*Wnt-Cre*$^{tg/+}$) and *Snrpb*$^{ncc\ +/-}$ pups stained with Alcian blue and Alizarin red **A**. Representative images of ventral views showing normal closed palate and normal bones in the head. The single mutant from group 2 with a bony palate cleft (yellow arrowhead) is shown. This embryo was also missing the tympanic ring and alisphenoid bone; the abnormally shaped basisphenoid bones can also be seen. In the second image, a group 1 mutant with a closed bony palate, but abnormal alisphenoid, basisphenoid and hypoplastic tympanic ring is shown. **B**. Representative images of mandibles of a wild type, control pup shows the normal shape and morphology of the coronoid, angular and condyloid processes. In the *Snrpb*$^{ncc+/-}$ mutant from group 2 (mut 1), a symmetrical but abnormal lower jaw is shown. In this same embryo, Meckel's cartilage was shortened, and the angular processes was missing. In a second mutant from group 2 (mut 2), an asymmetrical lower jaw is shown with Meckel's cartilage and the angular process missing on one side. **C**. Representative images of lateral views showing normal tympanic ring (arrow) and ossification in the head of a normal embryo. In the representative images of two group 2 *Snrpb*$^{ncc+/-}$ mutants the tympanic ring was absent in one and hypoplastic (arrow) in the second. In addition, the bones of the middle ear were abnormal with ectopic bones in the middle ear (white arrowhead) in one mutant and missing in the second. E=ear, Y=eye, BS=basisphenoid bone, AS=alisphenoid bone, PL=palatine, PPPL= palatal process of palatine, PMX=premaxilla, PPPMX=palatal process of premaxilla, PPMX=palatal process of maxilla, Tr=tympanic ring of ear, AP=angular process, CP=coronoid process, CNP=condyloid process, MC=Meckel's cartilage.

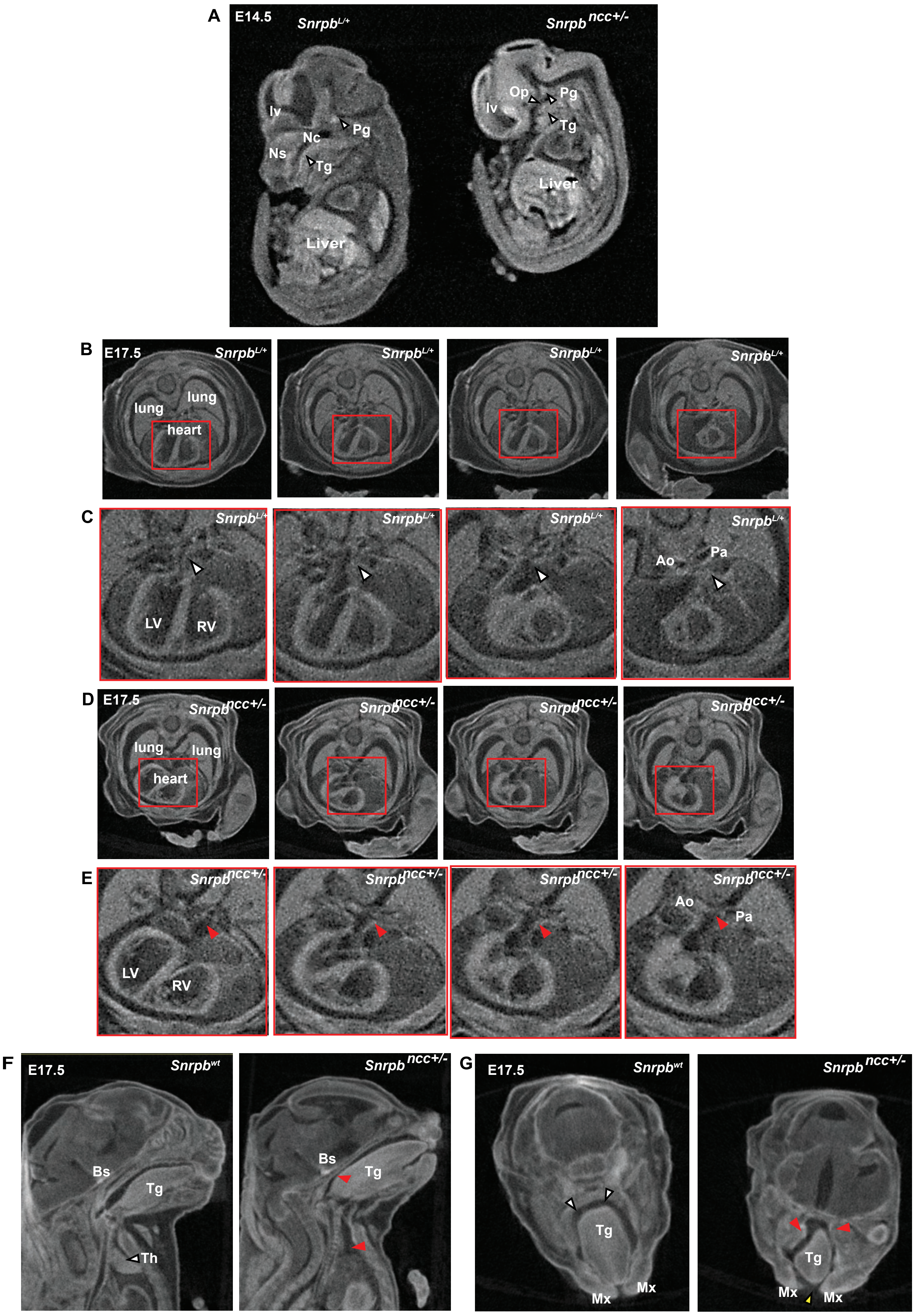

Fig. S4.

Disease Models & Mechanisms • Supplementary information

Fig. S4. Micro CT scan of *Snrpb*$^{ncc+/-}$ mutants show abnormal development of heart, brain, palate and thymus. **A**. Mid-sagittal MicroCT images of a control, (*Snprb*^{L}/+) embryo with normal morphological landmarks in the brain and face, and a *Snrpb*$^{ncc+/-}$ group 3 mutant with an enlarged lateral ventricle in the brain. The missing nasopharyngeal cavity and nasal septum can also be seen. **B**. and **C.** Transverse MicroCT images of the chest region of an E17.5 control embryo (*Snprb*^{L}/+). Panel **C**. show higher magnification of the heart (red box in panel B) from posterior to anterior (left to right of the panel), and the aorticopulmonary septum (white arrowhead) separating the aorta and pulmonary arteries in the control embryo (n=1). **D**. and **E**. Transverse MicroCT images of the chest and heart region of an E17.5 group 2 *Snrpb*$^{ncc+/-}$ mutant. **E.** in the higher magnification of the heart (red box in panel D) the aorticopulmonary septum is missing (red arrowhead). **F**. Mid-sagittal view of the same group 2 *Snrpb*$^{ncc+/-}$ mutant showing absence of the thymus (red arrowhead) which can be seen in the control embryo (white arrowhead). **G**. Sagittal views of a control and a group 2 E17.5 embryo shows the fused palatal shelves (white arrowheads) in the control, and clefts in the palate and the maxilla in the *Snrpb*$^{ncc+/-}$ mutant (red arrowheads). lv=lateral ventricle, Ns =nasal septum, Nc=nasopharyngeal cavity, Pg=pituitary gland, Tg=tongue, Op=oropharynx, LV=left ventricle, RV=right ventricle, Ao=aorta, Pa=pulmonary artery, Bs=basisphenoid bone, Th=thymus, Mx=maxilla

Disease Models & Mechanisms • Supplementary information

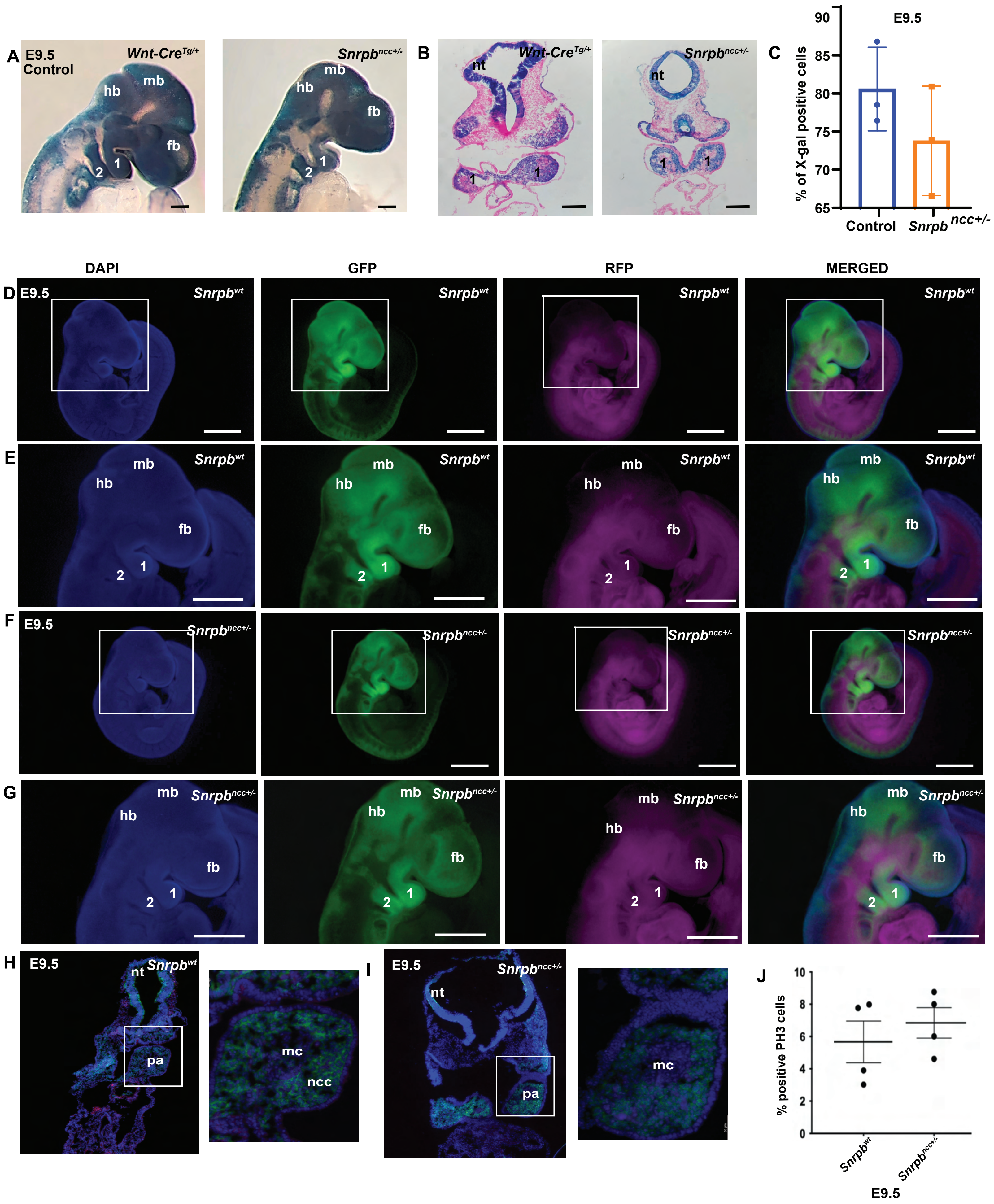

Fig. S5.

Disease Models & Mechanisms • Supplementary information

Fig. S5. Neural crest cells number and proliferation are not affected in E9.5 *Snrpb*$^{ncc+/-}$mutants. A. Representative images of an X-gal stained E9.5 control (*Wnt1-Cre*$^{Tg/+}$) and a group 2 *Snrpb*$^{ncc+/-}$ embryos. Fewer X-gal positive cells (blue) were found in the craniofacial region and the pharyngeal arches of the mutant. **B**. and **C**. Representative image of cryosection in a group 2 embryo and quantification of blue cells revealed a non-significant reduction in the percentage of X-gal positive cells in *Snrpb*$^{ncc+/-}$(n=3) embryos when compared to controls (n=3). **D**-**G**. Representative images of DAPI stained E9.5 control (*Snrpb*^{wt}) and group 1 *Snrpb*$^{ncc+/-}$ embryos carrying the mT/mG reporter. Green-fluorescence marks *Wnt1*-cre-expressing cells. **D.** and **F.** Lower magnification images of whole embryos. **E**. and **G**. Higher magnification of the craniofacial region (boxes in panel D and F) of control and mutant embryos, respectively, showing similar proportion of GFP^{+} cells in the mutant embryo **H**. and **I**. A magnified view of the pharyngeal arch of cryosectioned embryos (n=3) showing presence of GFP^{+} cells (green) in the pharyngeal arches of controls and mutants, respectively. **J** and **K**. Quantification of phosphohistone H3 positive cells in the craniofacial region revealed no significant difference between controls (*Snrpb*^{wt}) and mutants at E9.5 or E10.5. At E9.5, n=4 *Snrpb*$^{ncc+/-}$ mutants (3 group 1 and 1 group 2). At E10.5 n=5 controls and 5 *Snrpb*$^{ncc+/-}$ mutants (1 group 1 and 4 group 2). Error bars indicate standard error of mean (SEM) hb=hindbrain, mb=midbrain, fb=forebrain, 1,2=pharyngeal arch 1 and 2, respectively, pa=pharyngeal arch, nt=neural tube, ncc=neural crest cells, mc=mesenchymal core.

Disease Models & Mechanisms • Supplementary information

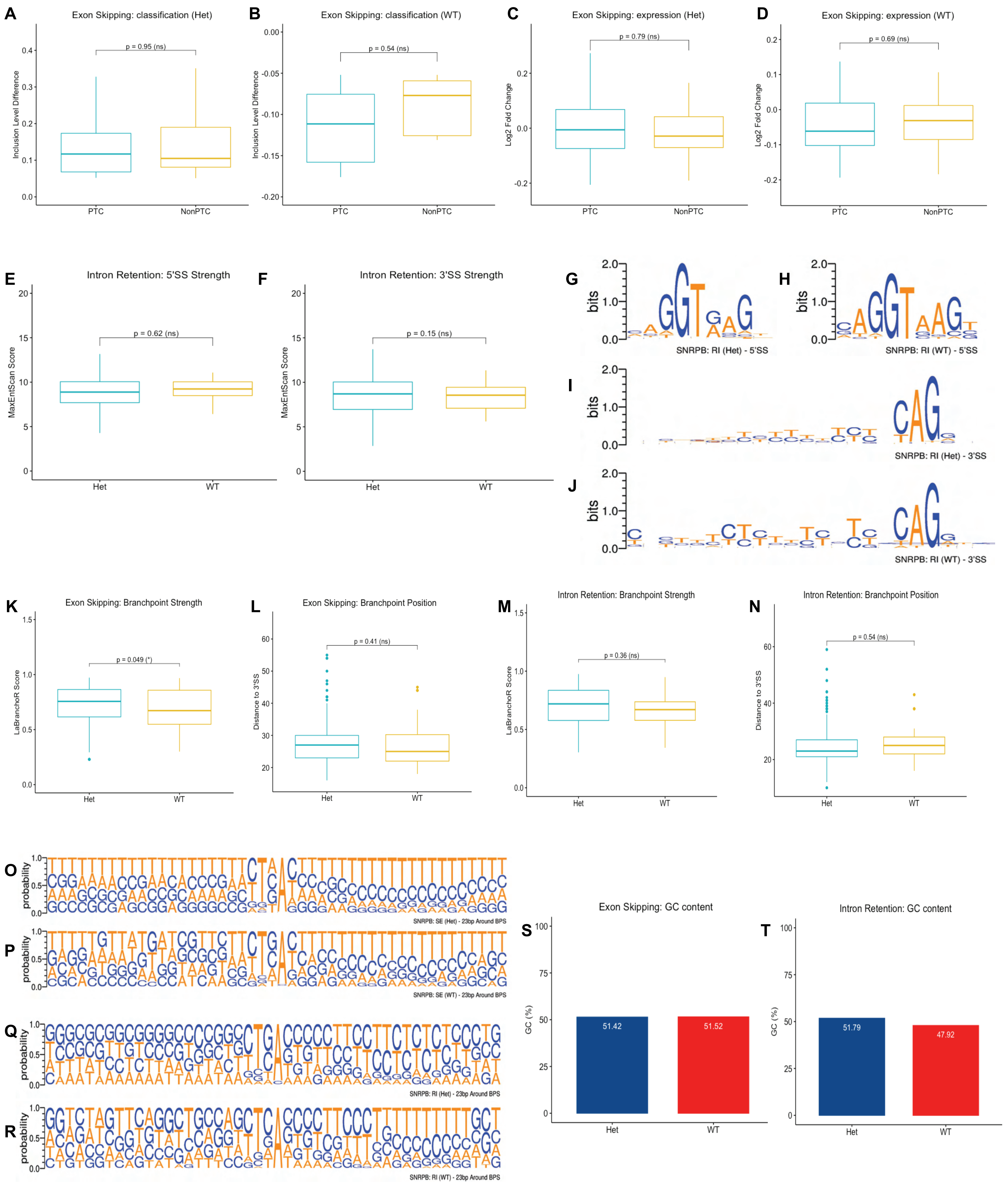

Disease Models & Mechanisms • Supplementary information

Fig. S6. Increased branch point strength might contribute to aberrant splicing in *Snrpb*$^{ncc+/-}$ mutants. A. and **B.** Presence of a premature termination codons (PTCs) in exons that are more skipped in mutant (**A**) and wild-type embryos (**B**) do not impact exon inclusion level. No significant difference was observed between (more skipped) PTC and Non-PTC exons. Similarly, **C.** and **D.** show no effect of PTCs in exons that are more skipped in mutant (**C**) and wild-type embryos (**D**) on the expression of genes carrying those exons. **E**. and **F**. Splice site weakness assessment (MaxEntScan scores): comparative analysis of the 5 'splice site strength (**E**) and 3' splice site strength (**F**) of introns that are more retained in mutant embryos and wild-type embryos, respectively. No significant difference was found in both analyses between the 2 conditions (Significance cut-offs, p-value <= 0.1[*] or p-value <= 0.05[**]), T-test. **G** and **H**. A 9-mer (3 exonic flanks and 6 intronic flanks around the 5'ss) consensus motif derived from introns that are more retained in mutant embryos (**G**) and wild-type embryos (H). **I** and **J**. A 23-mer (20 intronic flanks and 3 exonic flanks around the 3'ss) consensus motif derived from introns that are more retained in mutant embryos (**I**) and wild-type embryos (**J**). **K**. Highlights a significant difference in the strength of the BP sites (taking into account the LaBranchoR BP scores, 23bp upstream the 3'SS of exons that are more skipped in mutant embryos in comparison of those skipped in the wild-type embryos), T-Test. **L**. A mean comparison test (t-test) of the BP Distance from the 3'SS of the exons that are more skipped in mutant compared to the wild-type embryos. **M**. Highlights a mean comparison test (T-Test) of the LaBranchoR predicted BP Score from the introns that are more retained in mutant embryos in comparison to those more retained in the wild-type embryos, with no significant difference. **N**. A mean comparison test (T-Test) of the BP Distance from the 3'SS of the introns that are more retained in the mutant compared to the wild-type embryos. - Significance cutoffs used: p-value > 0.05 [ns] and p-value <= 0.05[*]. **O** and **P**. A (consensus) motif analysis of the branchpoint site (23bp around the LaBranchoR predicted branchpoint [BP]) from exons that are more skipped in mutant embryos (O) and wild-type embryos (P). **Q** and **R**. A (consensus) motif analysis of the branchpoint sites (23bp around the LaBranchoR predicted BPS) from introns that are more retained in mutant (Q) and wild-type embryos (R). **S** and **T**. show a GC content analysis (G+C nucleotide frequency analysis) of the 23bp sequences, upstream the LaBranchoR predicted BP of exons that are more skipped (S) and more retained (T) in mutant and wild-type embryos. A clear higher GC proportion in the mutant embryos is seen in case of retained intron.

Disease Models & Mechanisms • Supplementary information

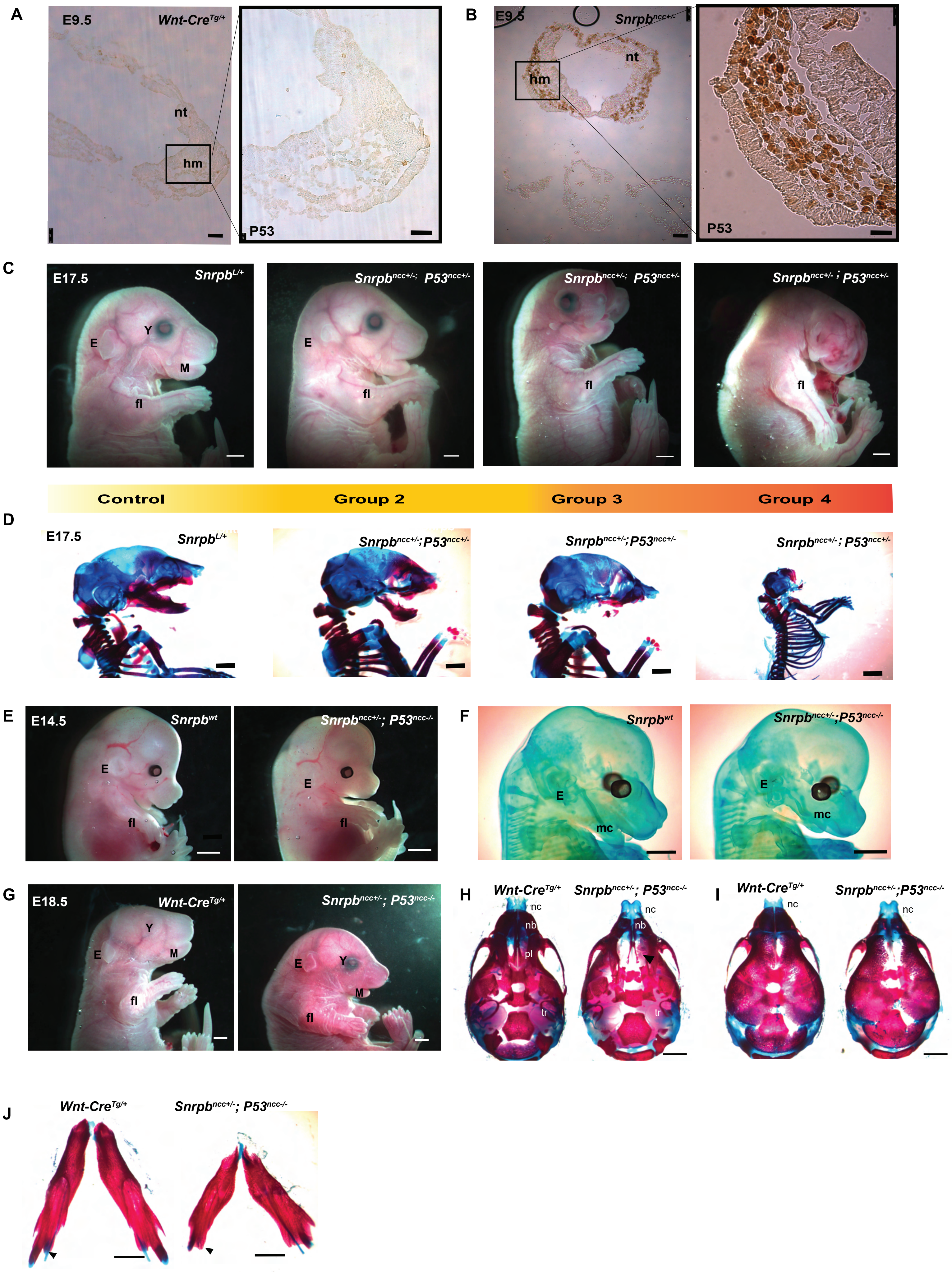

Disease Models & Mechanisms • Supplementary information

Fig. S7. Knockdown of P53 in the neural crest cells do not rescue craniofacial abnormalities in *Snrpb*$^{ncc+/-}$ mutants. A. and **B.** Representative images of sections of heads of E9.5 control (*Wnt1-Cre*$^{Tg/+}$) and *Snrpb*$^{ncc+/-}$ embryos after immunohistochemistry to detect P53 (brown). Increased nuclear P53 was found in mutants (n=3) when compared to controls (n=3). **C**. Similar range of phenotypic abnormalities was found in E17.5 *Snrpb*$^{ncc+/-}$ embryos heterozygous for *Trp53* when compared to *Snrpb*$^{ncc+/-}$ embryos (shown in Figure 1) **D**. Representative images of skeletal staining of E17.5 control (*Snrpb*$^{L/+}$) and *Snrpb*$^{ncc+/-}$; *P53*$^{ncc+/-}$ embryos showing that P53 heterozygosity in neural crest cells do not rescue craniofacial abnormalities in *Snrpb*$^{ncc+/-}$embryos. **E**. Representative images of E14.5 control (*Snrpb*$^{L/+}$) and *Snrpb*$^{ncc+/-}$; *P53*$^{ncc-/-}$ embryos showing micrognathia and hypoplasia of the head and outer ear in the mutant. **F** Representative images of E14.5 control (*Snrpb*$^{L/+}$) and *Snrpb*$^{ncc+/-}$; *P53*$^{ncc-/-}$ embryos after Alcian blue staining showing a hypoplasia of head and nasal cartilages and a curvy and shorter Meckel's cartilage in the mutant. **G**. Representative images of E18.5 control and *Snrpb*$^{ncc+/-}$; *Trp53*$^{ncc-/-}$ embryos. *Snrpb*$^{ncc+/-}$; *Trp53*$^{ncc-/-}$ (n=4) had microcephaly, a shorter snout and micrognathia. **H**. and **I**. Representative images of Alcian blue and Alizarin red stained skulls of E18.5 control and E18.5 *Snrpb*$^{ncc+/-}$; *Trp53*$^{ncc-/-}$ embryos. **H.** Cranial base view shows hypoplasia of the nasal bone, cleft palate (arrowhead) and hypoplasia of the tympanic ring in the *Snrpb*$^{ncc+/-}$; *Trp53*$^{ncc-/-}$ mutant. **I.** Top view of the calvaria shows reduced ossification of the frontal bone, reduced ossification, and reduced ossification of the nasal bone in the mutant. **J**. Representative images of the lower jaws of a control and a *Snrpb*$^{ncc+/-}$; *Trp53*$^{ncc-/-}$ mutants showing asymmetrical and abnormal development of the mutant mandible. Arrowhead in control indicates the angular process which is not discernable in the mutant (arrowhead). nt=neural tube, hm=head mesenchyme. E=ear, Y=eye, M=mandible, fl=forelimb, mc=Meckel's cartilage, nc=nasal cartilage, nb=nasal bone, tr=tympanic ring, pl=palate.

Disease Models & Mechanisms • Supplementary information

Table S1. Increased SE of craniofacial developmental genes in *Snrpb* $^{ncc+/-}$ mutants. Transcripts required for normal head/craniofacial development with a significant increase in skipped exons are not all predicted to result in PTC in *Snrpb*$^{ncc+/-}$.

Gene name	Skipped Exon	PTC	Phenotype	Constitutive exon
1. *Smad2*	Exon 3	No	Mandible hypoplasia (Nomura and Li, 1998)	No
2. *Loxl3*	Exon 2	No	Cleft palate, short and bent mandible (Zhang *et al.*, 2015)	No
3. *Ror2*	Exon 8	No	Midface hypoplasia, truncated Meckel's, middle ear defect (Schwabe, G.C. *et al.*, 2004)	Yes
4. *Nisch*	Exon 6	No	Short snout (Crompton, M. *et al.*, 2017)	Yes
5. *Pou2f1*	Exon 4	No	Abnormal nasal placode development when removed with *Sox2* (Donner, A.L. *et al.*, 2007)	Yes
6. *Rgl1*	Exon 3	No	Abnormal frontal bone, short snout, abnormal maxilla and mandibular morphology (Mouse Genome Informatics and the International Mouse Phenotyping Consortium, 2014)	Yes
7. *Frem1*	Exon 32	No	Midface hypoplasia, asymmetry, short snout (Vissers, L.E. *et al.*, 2011)	Yes
8. *Smc3*	Exon 5	No	Upturned snout (White, J.K. *et al.*, 2013)	Yes
9. *Pdpk1*	Exon 3	Yes	Abnormalities in the head, nasal cartilage (Lawlor, M.A. *et al.*, 2002)	Yes
10. *Rere* (*Atr2*)	Exon 4	Yes	Small pharyngeal arch (Zoltewicz, J.S. *et al.*, 2004)	Yes
11. *Mcph1*	Exon 13	Yes	Microcephaly (Gruber, R. *et al.*, 2011)	Yes
12. *Nf1*	Exon 56	Yes	Head hyperplasia, aorticopulmonary septal defect, heart defects (Brannan, C.I. *et al.*, 1994)	Yes
13. *Dyrk2*	Exon 2	Yes	Cleft palate (Yoshida, S. *et al.*, 2020)	No

Disease Models & Mechanisms • Supplementary information
